# Substantial light woodland and open vegetation characterized the temperate forest biome before *Homo sapiens*

**DOI:** 10.1126/sciadv.adi9135

**Published:** 2023-11-10

**Authors:** Elena A. Pearce, Florence Mazier, Signe Normand, Ralph Fyfe, Valérie Andrieu, Corrie Bakels, Zofia Balwierz, Krzysztof Bińka, Steve Boreham, Olga K. Borisova, Anna Brostrom, Jacques-Louis de Beaulieu, Cunhai Gao, Penélope González-Sampériz, Wojciech Granoszewski, Anna Hrynowiecka, Piotr Kołaczek, Petr Kuneš, Donatella Magri, Małgorzata Malkiewicz, Tim Mighall, Alice M. Milner, Per Möller, Małgorzata Nita, Bożena Noryśkiewicz, Irena Agnieszka Pidek, Maurice Reille, Ann-Marie Robertsson, J. Sakari Salonen, Patrick Schläfli, Jeroen Schokker, Paolo Scussolini, Vaida Šeirienė, Jaqueline Strahl, Brigitte Urban, Hanna Winter, Jens-Christian Svenning

**Affiliations:** ^1^ Center for Ecological Dynamics in a Novel Biosphere (ECONOVO) and Center for Biodiversity Dynamics (BIOCHANGE), Department of Biology, Aarhus University, Ny Munkegade 114, DK-8000 Aarhus C, Denmark.; ^2^ Section for Ecoinformatics and Biodiversity, Department of Biology, Aarhus University, Ny Munkegade 114, DK-8000 Aarhus C, Denmark.; ^3^ Department of Environmental Geography, CNRS UMR GEODE 5602, University Toulouse Jean Jaurès, Toulouse, France.; ^4^ Center for Sustainable Landscapes under Global Change (SustainScapes), Department of Biology, Aarhus University, Ny Munkegade 114, DK-8000 Aarhus C, Denmark.; ^5^ Center for Landscape Research in Sustainable Agricultural Futures, Department of Biology, Aarhus University, Ny Munkegade 114, DK-8000 Aarhus C, Denmark.; ^6^ School of Geography, Earth and Environmental Sciences, University of Plymouth, Plymouth, UK.; ^7^ CEREGE, CNRS, IRD, Europôle de l'Arbois, BP 80, F-13545 Aix-en-Provence, France.; ^8^ Faculty of Archaeology, Leiden University, Einsteinweg 2, 2333 CC, Leiden, Netherlands.; ^9^ Department of Geology and Geomorphology, University of Łódź, Narutowicza 88, 90-139 Łódź, Poland.; ^10^ Faculty of Geology, University of Warsaw, Warsaw, Poland.; ^11^ Department of Geography, University of Cambridge, Cambridge CB2 3EN, UK.; ^12^ Independent researcher, Soloviny str. 4-1-224, 117593, Moscow, Russia.; ^13^ Department of Geology, Lund University, Sölvegatan 12, SE-223 62 Lund, Sweden.; ^14^ Gymnasieskolan Knut Hahn, Blasius Königsgatan 27, 37232 Ronneby, Sweden.; ^15^ Mediterranean Institute of Marine and Terrestrial Biodiversity and Ecology, Aix-Marseille University, Marseille, France.; ^16^ Ontario Geological Survey, 933 Ramsey Lake Road, Sudbury, ON P3E 6B5, Canada.; ^17^ Instituto Pirenaico de Ecología, IPE–CSIC, Avda/Montañana 1005, 50059 Zaragoza, Spain.; ^18^ Polish Geological Institute, National Research Institute, Carpathian Branch, Skrzatów 1, 31-560 Kraków, Poland.; ^19^ Polish Geological Institute – National Research Institute, Marine Geology Branch, ul. Kościerska 5, 80-328 Gdańsk, Poland.; ^20^ Climate Change Ecology Research Unit, Faculty of Geographical and Geological Sciences, Adam Mickiewicz University Poznań, Bogumiła Krygowskiego 10, Poznań 61-680, Poland.; ^21^ Department of Botany, Charles University, Prague, Czechia.; ^22^ Dipartimento di Biologia Ambientale, University of Rome ‘La Sapienza’, Rome, Italy.; ^23^ Laboratory of Paleobotany, Department of Stratigraphical Geology, Institute of Geological Sciences, University of Wroclaw, Cybulskiego 34, 50-205 Wroclaw, Poland.; ^24^ Department of Geography and Environment, School of Geosciences, University of Aberdeen, UK.; ^25^ Department of Geography, Royal Holloway University of London, Egham, UK.; ^26^ Faculty of Natural Sciences, University of Silesia, Będzińska 60, 41-200 Sosnowiec, Poland.; ^27^ Faculty of Earth Sciences and Spatial Management, Nicolaus Copernicus University in Toruń, Lwowska 1, 87-100 Toruń, Poland.; ^28^ Maria Curie-Sklodowska University, Institute of Earth and Environmental Sciences, Al. Krasnicka 2 d, 20-718 Lublin, Poland.; ^29^ Department of Physical Geography and Quaternary Geology, Stockholm University, SE-106 91 Stockholm, Sweden.; ^30^ Department of Geosciences and Geography, University of Helsinki, Helsinki, Finland.; ^31^ Institute of Plant Sciences and Oechger Centre for Climate Change Research, University of Bern, Altenbergrain 21, 3013 Bern, Switzerland.; ^32^ TNO, Geological Survey of the Netherlands, Postbus 80015, 3508 TA, Utrecht, Netherlands.; ^33^ Faculty of Science, Department of Earth Sciences, Vrije Universiteit Amsterdam, Amsterdam, Netherlands.; ^34^ Institute for Environmental Studies, Vrije Universiteit Amsterdam, Amsterdam, Netherlands.; ^35^ Nature Research Centre, Institute of Geology and Geography, Akademijos 2, LT-08412 Vilnius, Lithuania.; ^36^ Landesamt für Bergbau, Geologie und Rohstoffe, Inselstraße 26, 03046 Cottbus, Germany.; ^37^ Leuphana University Lüneburg, Institute of Ecology, Lüneburg, Germany.; ^38^ Polish Geological Institute, 00-975 Warsaw, Poland.

## Abstract

The extent of vegetation openness in past European landscapes is widely debated. In particular, the temperate forest biome has traditionally been defined as dense, closed-canopy forest; however, some argue that large herbivores maintained greater openness or even wood-pasture conditions. Here, we address this question for the Last Interglacial period (129,000–116,000 years ago), before *Homo sapiens*–linked megafauna declines and anthropogenic landscape transformation. We applied the vegetation reconstruction method REVEALS to 96 Last Interglacial pollen records. We found that light woodland and open vegetation represented, on average, more than 50% cover during this period. The degree of openness was highly variable and only partially linked to climatic factors, indicating the importance of natural disturbance regimes. Our results show that the temperate forest biome was historically heterogeneous rather than uniformly dense, which is consistent with the dependency of much of contemporary European biodiversity on open vegetation and light woodland.

## Teaser

Landscape reconstruction using pollen data shows that European temperate forests were open and heterogeneous before modern humans.

## INTRODUCTION

The extent of vegetation openness in past European landscapes is widely debated (*1*–*4*). Uncertainties are especially acute in temperate forests, where accurate estimates are needed as baselines for ecosystem restoration. The traditional view is that closed-canopy forests, as the climax state of vegetation succession, would have dominated the temperate forest biome before increased human presence (*1*, *2*). In the past two decades, proxy-based reconstructions have challenged this view of European forests (*3*–*5*). Recent pollen-based reconstructions of past land cover in the Holocene [11,700 years before present (B.P.) to present] have shown that traditional comparisons of the percentage of arboreal to non-arboreal pollen strongly underestimate the cover of grass and heathland (*6*, *7*). In support of this finding, fossil records from habitat-specific Mollusca (molluscs) and Coleoptera (beetles, from the British Isles) indicated that open and semi-open vegetation dominated in the early- to mid-Holocene (11,700–6000 B.P.) (*8*) and during the Last Interglacial period [129–116 thousand years (ka) B.P.] (*9*), respectively. Therefore, rather than comprising exclusively closed forests, Europe was potentially a heterogeneous landscape that featured a mixture of closed, open, and semi-open vegetation, such as grassland, scrub, and wood-pasture–like vegetation (*4*, *10*). However, in the early Holocene, it is unclear how far open vegetation is an anthropogenic signal (*11*). The extent of vegetation openness before the impacts of *Homo sapiens*, in the temperate forest biome and Europe more broadly, remains poorly quantified.

Researchers have often considered the early to mid-Holocene, before the widespread adoption of agriculture, to be an appropriate reference point for prehuman vegetation structure (*2*, *3*, *5*). However, as a prehuman baseline for Europe, the early- to mid-Holocene is insufficient, primarily because of the impact of the arrival of *H. sapiens* (~54 ka B.P.) (*11*). While earlier humans, such as Neanderthals (*Homo neanderthalensis*), likely had localized effects on vegetation (*12*), there is evidence for the widespread use of fire by *H. sapiens* to shape vegetation during the Mesolithic (*13*). Furthermore, *H. sapiens* are likely to have reduced the density and distribution of large herbivores far more than previous hominins did (*12*, *14*). Large herbivores strongly influence vegetation openness (*15*). The global expansion of *H. sapiens* is associated with strong reductions in species richness and functional diversity of large herbivores, with particularly severe losses among larger species (*14*, *16*). These defaunation dynamics are likely to have reduced the ability of fauna to promote openness in landscapes. Before these losses, high megafaunal diversity was typical in Europe and worldwide for more than 20 million years (*17*). To understand pre–*H. sapiens* vegetation dynamics and their implications for the evolutionary adaptations of species, it is important to elucidate vegetation structure before the late-Quaternary faunal downsizing (*16*).

The Last Interglacial in Europe (Eemian) corresponds to Marine Isotope Stage 5e (129–116 ka B.P.) (*18*) and predated the expansion of *H. sapiens* into Europe (*19*). Some early human influence did exist (*20*), as Neanderthals were present throughout Europe (*21*), but they likely only influenced local vegetation structure, owing to low population sizes (*20*). Furthermore, despite different climate forcing, the Last Interglacial was characterized by climates comparable to those of the present (*22*). As a result, it presents a valuable opportunity to study vegetation openness in the absence of extensive human impact and with climatic characteristics similar to today. However, there are large gaps in our understanding of the vegetation cover during this period.

Pollen records represent the most direct and widely available empirical data for recreating past vegetation cover (*23*). The dominant vegetation of temperate Europe during the Last Interglacial period has been inferred by dividing pollen percentage diagrams into distinct zones based on dominant taxa (*24*, *25*). Four common “zones” are broadly identified as a unimodal pattern of vegetation succession in central and temperate regions (*24*, *26*). The first is the pioneer, Protocratic, *Pinus-Betula* (pine-birch) phase, in which rising temperatures and increasingly fertile soils supported light-demanding vegetation. High temperatures peaked during the temperate Mesocratic period, which is traditionally considered to be closed-forest dominated by *Quercus* (oak) and *Corylus* (hazel; early-temperate), followed by *Carpinus betulus* (hornbeam; late-temperate). Last, toward the end of the interglacial, leached soils and falling temperatures of the Oligocratic/Telocratic phase were correlated with *Picea* (spruce) dominating along with *Pinus* and *Abies* (fir) and increasing vegetation openness (*25*).

Vegetation openness during the Last Interglacial period has mostly been determined by comparing the raw percentages of arboreal pollen with non-arboreal pollen, which has indicated a scarcity of grassland and heathland and an overrepresentation of woody cover (*2*, *27*). However, the use of raw pollen percentages fails to account for the nonlinearity of the pollen-vegetation relationship (*27*). Furthermore, other proxies for vegetation reconstruction provide conflicting estimates. Small mammal assemblages suggest that mixed woodlands, including open grassy habitats, likely existed in West and Central Europe, with more open forest-steppe landscapes occurring in South and Northeast Europe (*28*). Beetle assemblage records from the British Isles similarly indicate a mixture of closed forests, wood pasture, and open vegetation (*9*). Last, fossil finds of many large grazing animals and megaherbivore diet indicators indicate the presence of mixed woodland and open habitats across Europe (*4*).

The pollen-vegetation relationship is influenced by spatial scale, basin size, differences in sedimentary archives, and taxonomic differences in pollen productivity and dispersal characteristics (*27*, *29*). The Regional Estimates of VEgetation Abundance from Large Sites (REVEALS) model corrects for biases caused by these factors and provides the regional vegetation composition and land cover within a 1° × 1° area (*27*). The REVEALS model has been extensively validated using both modern and historical analogs (see Materials and Methods) (*30*–*32*). Pollen-based REVEALS reconstructions of vegetation openness over the Holocene were produced for 1° × 1° grid cells across Europe (*7*). However, REVEALS has only been used to reconstruct vegetation of the Last Interglacial period at single sites (*33*) and not at the continental scale.

In this study, we applied REVEALS to a large dataset of Last Interglacial pollen records across Europe. We assessed vegetation openness in the European temperate forest biome, as well as adjoining biomes, before the arrival of *H. sapiens*. To elucidate the processes controlling vegetation structure, we evaluated the extent to which climatic and topoedaphic factors explain the variation in pre-anthropogenic vegetation openness across Europe and within the temperate forest biome. Our study provides insights into the state of the temperate forest biome before modern humans and contributes to the long-standing “open” versus “closed” vegetation debate in Europe. Our results have important implications for our understanding of the evolutionary ecology of Europe’s native biota as well as for restoration and rewilding efforts within this biome and across the continent.

## RESULTS

### Europe-wide scale

**Fig. 1.**
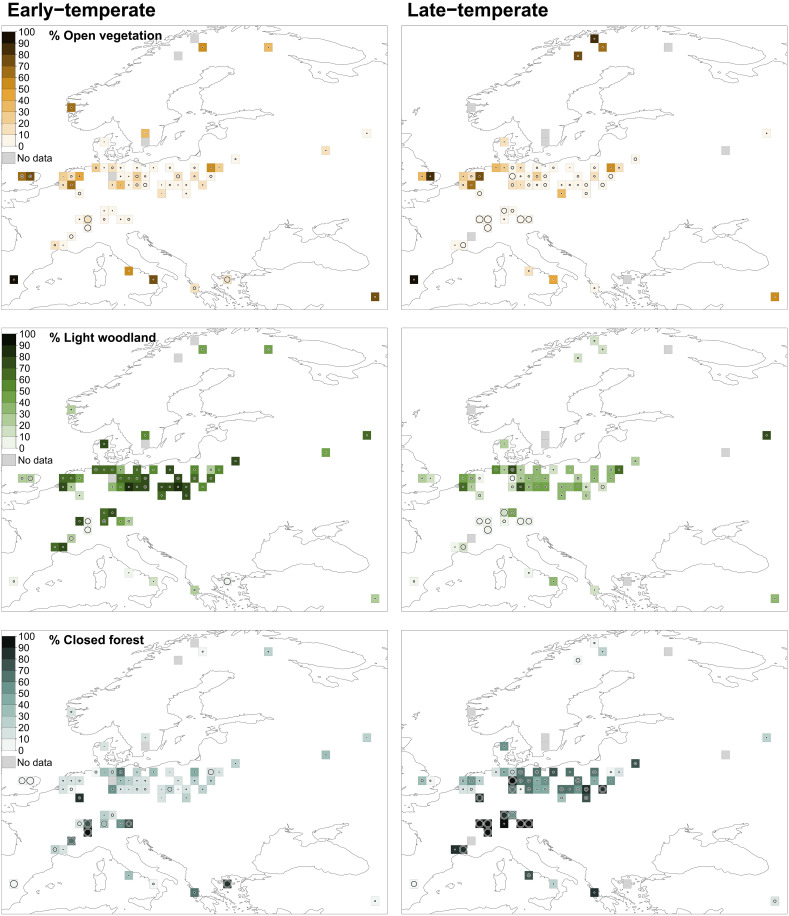
REVEALS estimates. Open vegetation (**top**; herbaceous and heathland taxa) light woodland (**middle**; shade-intolerant and intermediate taxa) and closed forest (**bottom**; shade-tolerant taxa) in the early-temperate (left) and late-temperate (right) periods. Each square is a regional grid cell of 1° × 1°. Darker colors show greater cover. White/black circles inside each grid cell represent the coefficient of variation (SE/REVEALS estimate). When SE ≥ REVEALS estimate, the circle fills the entire grid cell, and the estimate is considered unreliable.

Our results showed that, before the arrival of *H. sapiens*, highly heterogeneous vegetation was widespread in Europe (Fig. 1). Taxa indicating open and light woodland vegetation were strongly represented alongside the closed forests of shade-tolerant trees. In the early-temperate period, open vegetation represented an average of 21% {95% confidence interval (CI) [14.8, 26.2]} of the vegetation cover, with light woodland taxa representing an additional 53% (95% CI [47.0, 58.7]). We found that 16% of the grid cells contained open vegetation over more than 50% of their area (*n* = 10; Fig. 1). Low levels of open-vegetation taxa, between 0 and 10%, were found in 48% of the grid cells (*n* = 30; Fig. 1).

In the late-temperate period, open vegetation represented an average of 19% (95% CI [12.6, 25.4]) of the vegetation cover, with light woodland taxa representing an additional 28% (95% CI [23.3, 32.6]; Fig. 1). We found that 15% of the grid cells contained more than 50% open vegetation (*n* = 9; Fig. 1). The late-temperate period had more grid cells with less than 10% open vegetation taxa (58% of grid cells, *n* = 34, compared to 48% in the early-temperate period, *n* = 30; Fig. 1).

During both time periods, Poaceae (grasses) and Cyperaceae (sedges) were the dominant open vegetation taxa, and *Corylus* was the dominant light-woodland taxon (table S1). Other common taxa in the two categories were *Artemisia* (mugworts), Amaranthaceae/Chenopodiaceae (gooseworts and relatives), Ericaceae (various heathers and relatives), *Rumex acetosa* type (sorrel), *Calluna vulgaris* (common heather), *Betula*, *Pinus*, *Salix* (willow), and *Taxus baccata* (yew; data S1).

### Temperate forest biome

**Fig. 2.**
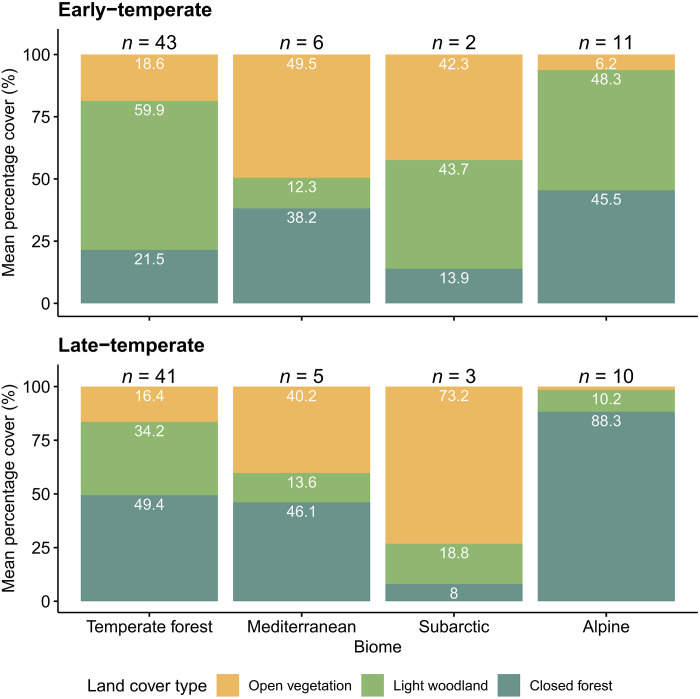
Mean percentage land cover type per biome. Mean cover (%) of open vegetation (yellow), light woodland (green), and closed forest (blue) in the temperate forest, Mediterranean, subarctic, and Alpine biomes in the early-temperate (**top**) and late-temperate (**bottom**) periods of the Last Interglacial.

In the temperate forest biome (oceanic and continental sites that are not in the “Alpine” region; see fig. S1), open and light woodland taxa combined represented 79% (95% CI [74.0, 83.0]) of the vegetation, on average, in the early-temperate period and 51% (95% CI [43.3, 57.9]) in the late-temperate period (Fig. 2). In the early-temperate period, open taxa represented an average of 19% (95% CI [12.8, 24.5]) of the vegetation, while light woodland taxa represented an additional 60% (95% CI [54.4, 65.3]; Fig. 2). We found that 12% of the grid cells contained more than 50% open vegetation (*n* = 5), whereas 49% of the grid cells contained 0 to 10% open vegetation (*n* = 21; Fig. 1). In the late-temperate, open taxa represented an average of 16% (95% CI [10.2, 22.5]) of the vegetation, and light woodland taxa represented an additional 34% (95% CI [29.0, 39.4]; Fig. 2). We found that 10% of the grid cells contained greater than 50% open vegetation (*n* = 4), whereas the number of grid cells with less than 10% open vegetation increased slightly to 56% (*n* = 23; Fig. 1). During both periods, the most open sites tended to occur in oceanic Europe. However, multiple exceptions existed and did not follow any spatial pattern, nor were they assigned to a particular biome (Fig. 1).

### Alpine region

The Alpine region (fig. S1) contained relatively little open vegetation (Figs. 1 and 2). All grid cells contained less than 20% of open vegetation taxa in the early-temperate period (*n* = 11) and less than 10% of open vegetation taxa in the late-temperate period (*n* = 10; Fig. 1). The presence of light woodland taxa varied. In the early-temperate period, light woodland taxa represented between 0 and 80% of the vegetation. The late-temperate period contained a much lower percentage of light woodland taxa, with most grid cells containing less than 20% (*n* = 9; Fig. 1). Most of the region was closed forest, particularly during the late-temperate period, when all grid cells contained 90 to 100% closed forest vegetation (Fig. 1). In most grid cells, the SE was greater than the REVEALS estimate for closed forests, raising uncertainty regarding the reliability of the results (Fig. 1 and data S2). However, the grid cells in this region were unanimous in their findings. *Picea*, *Abies alba* (silver fir), *Corylus*, and *Quercus* were dominant in the early-temperate period, indicating a mixed closed and light woodland landscape. *A. alba* was the dominant taxon in the late-temperate period, indicating a closed-forest dominated landscape.

### Other biomes

In the subarctic and Mediterranean biomes (fig. S1), open vegetation taxa dominated, but vegetation openness was highly variable. In the Mediterranean, during the early-temperate period, the six grid cells contained between 10 and 100% open vegetation, with an average openness of 50% (95% CI [16.6, 73.7]; Figs. 1 and 2). In the subarctic, both grid cells contained 30 to 50% open vegetation (Fig. 1). The late-temperate period showed a similar variation in openness; in the Mediterranean, grid cells had between 0 and 100% open vegetation (mean = 40%; 95% CI [8.4, 51.3]), whereas the three subarctic sites contained 60 to 90% open vegetation (Figs. 1 and 2). A high level of light woodland taxa was present during the early-temperate period in the subarctic (40 to 50% cover), which dropped to less than 20% in the late-temperate period (Fig. 1). In the Mediterranean, light woodland taxa represented between 0 and 30% of the vegetation in the early-temperate period and between 0 and 40% in the late-temperate period (Fig. 1).

### Drivers of vegetation openness

#### Continental scale

The full beta regression model included the following predictors: mean temperature of the warmest quarter (°C), precipitation of the driest month (millimeters), degree of continentality (the difference between the mean temperature of the warmest quarter and coldest quarter; °C), SD of elevation (terrain roughness; meters), occurrence in the Alpine region (1) or outside it (0), and time window (early-temperate and late-temperate). The model explained 29.9% of the variation in the data [pseudo coefficient of determination (*R*^2^)].

**Fig. 3.**
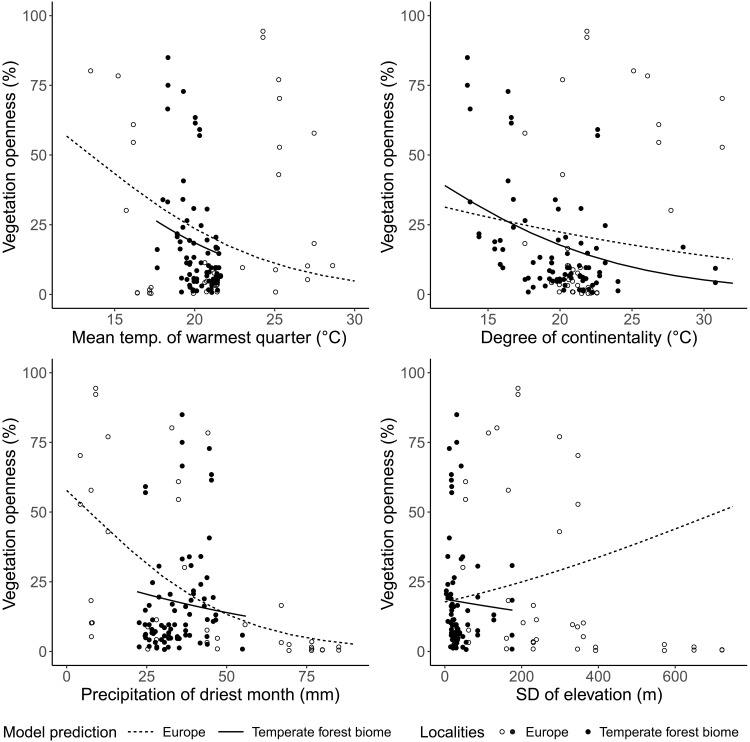
Beta regression predictions for variables with significant effects: Mean temperature of the warmest quarter (°C), degree of continentality (°C), precipitation of driest month (millimeters), and SD of elevation (meters). Two scales are shown: Europe (*n* = 118; all grid cells across both time windows; solid + unfilled points, and dashed lines), and the temperate forest biome (*n* = 82; oceanic and continental grid cells, excluding Alpine group, across both time windows; solid points and lines).

The precipitation of the driest month and mean temperature of the warmest quarter had the strongest effect on vegetation openness (estimate = −0.044, *P* < 0.001; and estimate = −0.181, *P* < 0.001, respectively; Fig. 3). The negative effect of temperature was largely driven by high openness and low temperatures in the subarctic, and the effect of precipitation was driven by high openness and low precipitation in the Mediterranean. There was moderate evidence that degree of continentality decreased vegetation openness, with openness increasing toward more oceanic conditions (estimate = −0.057, *P* = 0.045; Fig. 3). There was moderate evidence that open vegetation cover was affected by terrain roughness (estimate = 0.002, *P* = 0.011; Fig. 3). Last, a pairwise comparison of the estimated marginal means from the beta regression model revealed no difference in vegetation openness between the early-temperate and late-temperate periods (estimate = 0.028, *P* = 0.273; fig. S2).

#### Temperate forest biome

The full beta regression model for the temperate forest biome explained 22.5% of the variation in the data (pseudo *R*^2^). Increasing continentality was linked to decreasing vegetation openness to a strong degree (estimate = −0.137, *P* = 0.003; Fig. 3). No other explanatory variables had significant effects (Table 1 and fig. S2).

**Fig. 4.**
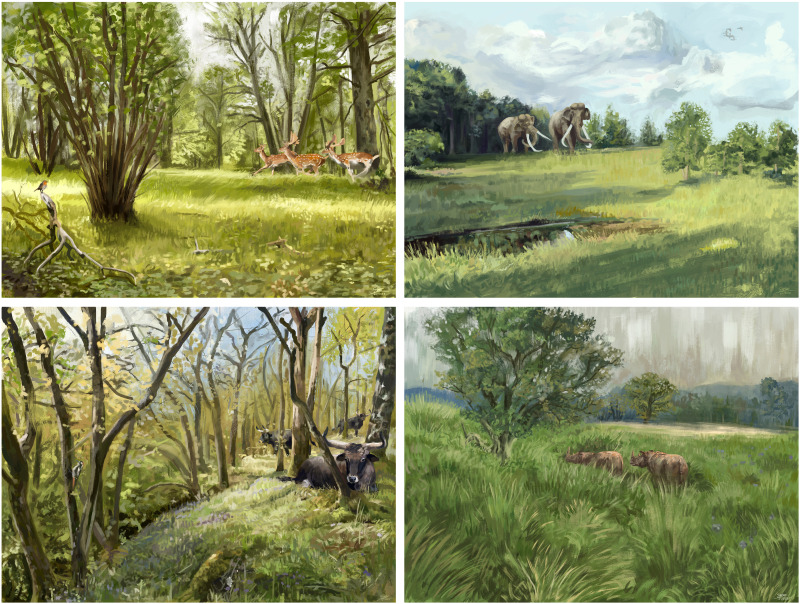
Palaeoartistic reconstructions of Last Interglacial landscapes in the European temperate forest biome, consistent with our pollen-based estimates of vegetation structure. Typical Last Interglacial fauna are shown, such as the extinct straight-tusked elephant (*Palaeoloxodon antiquus*), an extinct rhinoceros (*Stephanorhinus kirchbergensis*), and aurochs (*Bos primigenius*, the extinct wild form of contemporary domestic and feral cattle), alongside common extant species: fallow deer (*Dama dama*), a great spotted woodpecker (*Dendrocopos major*), a European robin (*Erithacus rubecula*), and greylag geese (*Anser anser*). (**Top left**) Early-temperate period: Light woodland, including a mix of taller trees and the shrub hazel (*Corylus avellana*), and grass-dominated open vegetation. (**Top right**) Early-temperate period: Open, grassy vegetation interspersed with light woodland and bordering closed forest with shade-tolerant trees. (**Bottom left**) Late-temperate period: Light woodland, denser forest with frequent hornbeam (*Carpinus betulus*), and some open vegetation (front). (**Bottom right**) Late-temperate period: Open grass- and sedge-dominated vegetation with free-standing deciduous oaks (*Quercus robur*), with more closed tree stands in the background. Illustrator: Brennan Stokkermans.

**Table 1.** Beta regression model output for all explanatory variables. Estimates and *P* values are given for all explanatory variables for Europe (all grid cells) and grid cells in the temperate forest biome (oceanic and continental grid cells, excluding Alpine group).

Coefficient	Estimate		*P* value	
	Europe (all grid cells)	Temperate forest biome	Europe (all grid cells)	Temperate forest biome
Mean temperature of the warmest quarter (°C)	−0.181	−0.189	0.001	0.164
Mean precipitation of the driest month (mm)	−0.044	−0.019	4.40 × 10^−5^	0.298
Degree of continentality (°C)	−0.057	−0.137	0.045	0.003
SD of elevation (m)	0.002	−0.002	0.011	0.569
Time window (early/late-temperate)	−0.196	−0.133	0.271	0.487
Alpine (0, 1)	−0.593		0.147	

#### Alpine region

Including all grid cells (*n* = 66) in the beta regression model revealed that the Alpine sites had a moderate negative association with vegetation openness (estimate = −0.827, *P* = 0.029; fig. S3). However, after removing the outlier in Southern Norway (see Materials and Methods), we found no effect (fig. S2).

### Robustness assessment

Multiple comparisons of means using Tukey contrasts revealed that the SEs were significantly higher for closed vegetation than for open vegetation (estimate = −18.82, *P* < 1 × 10^−4^) or light woodland vegetation (estimate = −15.58, *P* < 1 × 10^–4^; fig. S4). Furthermore, the SEs for open vegetation cover did not vary greatly across the openness levels (fig. S5). The REVEALS estimates for open vegetation were unreliable for only three grid cells (Fig. 1; see Materials and Methods). The REVEALS estimates were unreliable in one grid cell for light woodlands and 15 grid cells for closed vegetation (Fig. 1). All SEs are provided in data S2.

We found no effect of wetland indicators on vegetation openness. A pairwise comparison of the estimated marginal means from beta regression modeling showed no difference in vegetation openness between samples taken from bogs and lakes (estimate = −0.002, *P* = 0.950; see also fig. S6). We found no relationship between vegetation openness and the wetland taxa *Salix* (estimate = 11.127, *P* = 0.213) and *Alnus* (alder; estimate = −0.627, *P* = 0.919). Across Europe (all grid cells), we found moderate evidence for a relationship between lake size and vegetation openness (estimate = 4.499 × 10^−5^, *P* = 0.024). However, this relationship was driven by the grid cell from Lake Van, Turkey, which is the largest lake in the dataset (table S2). When we excluded this grid cell, we found no evidence of a relationship (estimate = −6.020 × 10^−5^, *P* = 0.353).

## DISCUSSION

Our analysis of vegetation openness in Europe before *H. sapiens* revealed three principal findings. First, open and light woodland vegetation were common in the temperate forest biome during the Last Interglacial. Second, vegetation cover was highly variable, with the proportion of open vegetation varying widely across the entire range. Together, these findings suggest that, before *H. sapiens*, the European temperate forest biome was a heterogeneous woodland landscape with widespread but varied open and light woodland vegetation (Fig. 4). Last, variation in open vegetation cover could only be partially explained by climatic and topoedaphic variables. Although the mean temperature of the warmest quarter, precipitation of the driest month, and degree of continentality had some effects on open vegetation cover, it is likely that other processes also played important roles in shaping European landscapes before modern humans.

Our continental-scale analysis supports a growing body of local-level, proxy-based work. The presence of grasslands, meadows, and other open vegetation have been indicated by plant macrofossil, mollusc, and beetle records (*4*, *8*, *9*); large herbivore diet analyses (*34*); and the presence of forb taxa that characterize grasslands and disturbed soils, such as *Artemisia*, Amaranthaceae/Chenopodiaceae, and *R. acetosa* (data S1) (*20*). Such findings have provided useful indications of open vegetation during the Last Interglacial period but have previously conflicted with findings from pollen records. For example, in the British Isles, Coleoptera assemblages indicated the presence of up to 55% wood-pasture landscapes as well as open and closed habitats in the Last Interglacial period (*9*). In Central Europe, the mammalian record indicated a mosaic environment of forested and open vegetation, based on the frequent occurrence of *Equus ferus* (wild horse), *Bison* spp. (bison), and *Bos primigenius* (aurochs) (*35*). Furthermore, analyses of small mammals revealed diverse faunal compositions indicative of diverse habitats (*28*). A considerable number of open landscape inhabitants (e.g., field voles *Microtus agrestis* and *Microtus arvalis*) as well as forest-dwelling species [e.g., *Apodemus sylvaticus* (wood mouse) and *Myodes glareolus* (bank vole)] were present across Europe during the Last Interglacial period (*28*). Our results present an important step toward resolving the contradictions between the floral- and faunal-based estimations of vegetation structure during the Last Interglacial period.

The high abundance of *Corylus* and deciduous *Quercus* (hereafter *Quercus*) in the light woodland category supports the presence of semi-open landscapes and is indicative of ongoing disturbance regimes. *Corylus* and *Quercus* were particularly visible during the early-temperate period (Fig. 1 and table S1), as these taxa are moderately light-demanding and grow opportunistically in open or light woodland areas and areas of recent disturbance (*36*, *37*). We expected high levels of *Quercus* and *Corylus* during the early-temperate because this pattern is characteristic of many of the Last Interglacial pollen diagrams (*24*). However, previous studies concluded that the temperate forests were *Quercus* dominated, with *Corylus* present to a lesser degree (*24*). Our REVEALS model estimated greater percentages of *Corylus* than *Quercus* during the early-temperate period. Although partial *Corylus* dominance after the *Quercus* phase of the temperate period has been shown previously (*38*), our findings suggest a much greater role for *Corylus*. *Corylus* dominance would be consistent with scrub woodland dependent on ongoing disturbance, although *Corylus* may also thrive under the canopy of lightly shaded trees, such as *Quercus* and *Fraxinus* (ash) (*37*). Furthermore, *Quercus* regeneration, from seed dispersal to recruitment, occurs mostly in dynamic, heterogeneous landscapes subjected to disturbances from grazing animals and fire, for example (*36*). Both *Corylus* and *Quercus* fail to regenerate under a dense canopy (*36*, *39*) and both taxa persisted for millennia through the temperate period (Fig. 1 and table S1). Although less common, the continued presence of *Corylus* and *Quercus* in the late-temperate period is consistent with a heterogeneous landscape with varied open elements, suggesting the presence of ongoing disturbances.

The variability in open vegetation across Europe raises questions about the drivers of openness because environmental and climatic factors only partially explain the distribution of vegetation openness. In focusing our analysis on the temperate forest biome, we found that vegetation was more open in the milder oceanic grid cells (Figs. 1 and 2). From a climatic perspective, this relationship is counterintuitive because tree dominance is expected under milder temperate conditions (*4*). Furthermore, we found little evidence of any effects of other climatic or environmental variables in this biome (fig. S2). It is possible that other environmental variables, such as soil type, played a role in driving open vegetation (*40*). However, soil type is difficult to assess for the Last Interglacial period, and comparisons to modern records are inadequate, given the transformation of relief and thus soil formation, structure, and texture following glacial cycles (*41*). Except for the Alpine group, the temperate biome grid cells revealed no clear spatial pattern of vegetation openness and no pattern likely to match any considered environmental gradient. Climate-linked openness is often mediated by disturbance factors (*42*). Furthermore, under mild temperate conditions today, trees tend to dominate via succession in the absence of the active restoration of disturbance regimes (*43*), even on poor soils (*44*). Therefore, we propose that disturbance agents must have influenced the presence of open and light woodland vegetation, with potentially stronger effects under more oceanic climates.

The presence of open and light woodland taxa suggests ongoing vegetation disturbance. A plausible candidate is the rich megafaunal community of Europe during the Last Interglacial (*15*, *35*). Large herbivores are ecological engineers capable of altering vegetation at the landscape scale (*17*). Their large body size requires the consumption of large quantities of plant biomass, which further affects vegetation through trampling, rooting, and debarking, as well as through seed dispersal and biogeochemical cycling (*17*). The effects of large herbivores on vegetation structure and wider ecosystem functioning have been well researched in recent years, especially relating to the ecosystem-wide effects of reintroductions (*45*). In modern European systems, large free-living herbivores can have considerable and lasting effects on vegetation composition and structure, for example, by generating or maintaining open and semi-open vegetation (*46*). Furthermore, their effects might be stronger under mild, oceanic climates, where population sizes are less constrained by cold and drought (*46*). Compared to present-day Europe, the Last Interglacial period was home to a considerably greater number of larger-bodied herbivores (*16*), including elephants and other megaherbivores with strong effects on vegetation structure (*15*, *47*). Our high openness estimates for England are consistent with previous beetle-based estimates, which also indicate high large herbivore abundances (*9*, *48*) at a level sufficient to generate open vegetation on fertile wetland adjacent soils in Western Europe today (*46*). In the present study, closed forest vegetation was more abundant in the Alpine region (Figs. 1 and 2). It is possible that, because of lower accessibility (*49*), larger herbivores were not as prevalent in this mountainous region and altered vegetation structure more in lowland regions (*4*, *9*). However, terrain roughness, a measure hypothesized to reflect herbivore accessibility (*49*), had a moderate positive effect on vegetation openness (Fig. 3). Further research is required to understand this relationship.

Large herbivores may alter forests beyond promoting vegetation openness, such as by affecting the structure and species composition of the closed vegetation community (*15*). Such effects might explain the expansion of *C. betulus* in the late-temperate period. *C. betulus* is one of the few dominant European tree species able to develop a “cage” architecture when exposed to browsing, allowing it to grow out of reach of herbivores (*50*). In addition, it has tough wood, a strong resprouting ability, and a folded trunk morphology that should protect against debarking. Consequently, *C. betulus* survives severe herbivory regimes more readily than other dominant European tree species, but especially under high light conditions (*50*). This adaptation challenges the view that abiotic drivers alone influence forest structure, even when forests are denser, as in the late-temperate period (Fig. 1). Our findings support the presence of sunlit conditions that could enable *C. betulus* survival and eventual dominance (table S1).

It is possible that fire regimes play a role in the unexplained patterns of vegetation openness. Feedback between fire and fire-prone grassy vegetation maintains open landscapes in some ecosystems (*51*). In boreal and Mediterranean ecosystems, fire disturbance is an important part of vegetation dynamics (*52*) and could contribute to the higher percentages of vegetation openness found in these regions. Moist temperate regions are often considered to have low fire frequencies and severity because broadleaf deciduous trees generally have high leaf moisture and little flammable material (*52*). Furthermore, a strong role of fire would not explain openness toward oceanic conditions, which are less fire-prone. Moreover, in the British Isles, fires were infrequent during the Last Interglacial period (*9*). However, quantifying the role of fire in the rest of the temperate forest biome during the Pleistocene interglacial periods is a promising avenue for future research, particularly given megafauna-fire interactions and the widespread consequence of herbivore extinction on global fire regimes (*53*). Furthermore, other disturbances, such as agents of abnormal intensity (floods, avalanches, storms, and landslides), are likely to have played a role in opening vegetation in some settings (*4*). The roles of these abiotic stochastic disturbances in interglacial ecosystems provide interesting future research opportunities.

Traditionally, closed-canopy forests are believed to have dominated the temperate forest biome before modern humans (*2*, *26*). Our findings show that European forests included substantial open and light woodland elements and suggest an important role for processes that maintain open habitats. This may have important implications for European biota and particularly for rarer species that depend on open, intermediate, and disturbed landscapes (*54*, *55*). Consequently, common approaches to restoration, such as tree planting, risk creating unfavorable habitats for biodiversity that has evolved in heterogeneous landscapes (*5*, *56*). Trophic rewilding and other approaches aimed at restoring natural disturbance factors may be better suited for restoring European forest biomes because they directly promote processes that increase habitat heterogeneity (*57*). Because of the value in understanding the structure of a biome in conservation and restoration, we advocate for a reimagining of the temperate forest biome to reflect the substantial open vegetation and light woodland present.

## MATERIALS AND METHODS

We focused our data collection and analyses on the temperate forest biome because we were explicitly interested in the vegetation openness of this bioclimatic region. We defined the temperate forest biome as an oceanic or continental climate zone traditionally considered dominated by temperate deciduous broad-leaved or mixed deciduous broadleaf-evergreen conifer forests (fig. S7 and table S3) (*58*). In addition, we collected pollen data from the adjoining subarctic and Mediterranean biomes to assess European vegetation cover more broadly and to further our understanding of the drivers of vegetation openness (fig. S7 and table S3). We focused on the temperate period of vegetation development during the Last Interglacial period, as it reflects the climatic optimum (*24*) and maximum vegetation biomass development (*26*). Notably, open vegetation often characterizes the beginning and end of interglacials in pollen diagrams due to the low soil quality and temperatures preceding and following glacial periods (*26*). Therefore, exploring openness in the central temperate period is most comparable to current conditions, both in terms of climate and positioning within an interglacial period.

### Pollen data collection and preparation

We collected 96 European pollen records from the European Pollen Database (www.europeanpollendatabase.net/), Pangaea (www.pangaea.de/), Neotoma (www.neotomadb.org/), and individual pollen data contributors (fig. S1 and table S2) and applied the vegetation reconstruction method REVEALS (*27*). The REVEALS model reconstructs vegetation cover regionally. This is achieved by quantifying background pollen from one or more sites to produce regional vegetation for a 1° × 1° area (*27*). REVEALS overcomes the nonlinearity of the pollen-vegetation relationship by accounting for relative pollen productivity (RPP), dispersal, and deposition differences between taxa (*27*). The model has been extensively tested and validated at sites across Europe (*59*, *60*) and North America (*61*), as well as at the European scale (*62*). Empirical testing against modern (*59*, *60*, *62*) and historical (*31*) analogs has shown that REVEALS improves the accuracy of vegetation reconstruction considerably compared to that using pollen proportions alone. Last, REVEALS is robust to variations in site selection, sampling design, and parameter values (*63*) and is considered a valuable tool for reconstructing past landscapes in different settings and environments, including small sites (*32*), floodplains (*64*), and mountainous regions (*31*).

Because radiocarbon dating was not possible for our study period, we selected pollen records that were dated to the Last Interglacial period based on litho- and/or bio-stratigraphical evidence (*65*). This is considered a robust approach for the Last Interglacial, as, in Europe, the Last Interglacial follows a very distinct, widely acknowledged pattern of vegetation succession by the dominant taxa, with the most closed vegetation phases occurring in the mesocratic/temperate phase (*24*, *26*). To avoid issues of interglacial non-synchronicity across Europe, we used dominant vegetation taxa to classify the Protocratic, Mesocratic (temperate), and Telocratic periods based on defined pollen zones of the Last Interglacial (*24*, *25*) following Lang’s protocol (*24*). Therefore, we examined vegetation openness in the *Quercus/Corylus*-dominated (first half of the Mesocratic: early-temperate) or *C. betulus*–dominated (second half of the Mesocratic: late-temperate) periods, rather than at specific times.

We implemented REVEALS using the protocol of Githumbi *et al.* (*7*) on the basis of the LRA R package (*66*). The REVEALS model uses pollen count data, RPP estimates, and pollen fall speed to reconstruct regional vegetation cover for each taxon in each time slice (Supplementary Materials). The REVEALS model is applied to lake and bog sites separately within each 1° × 1° grid cell and combines results, from several sites when available, to produce a single mean percentage cover estimate (data S1) and mean SE (data S2) for each RPP taxon per grid cell. Site locations with respect to their grid cells are available in the Supplementary Materials (fig. S8). The assumptions of the REVEALS model were presented by Sugita (*27*). We calculated the mean percentage cover of each plant functional and land-cover type by summing the mean percentage cover of each associated RPP taxon (Table 2) and averaged these values across all grid cells (*n* = 66) to provide Europe-wide estimates of vegetation openness. REVEALS calculates the uncertainty, using the delta method (*67*), as the SEs derived from the sum of the within- and between-site variations in the grid cell (data S2) (*7*). We also calculated the coefficient of variation (SE/REVEALS estimate) to report SEs, as shown in Fig. 1. We considered SEs to be unreliable when they were greater than the REVEALS estimate. We identified three unreliable grid cells for open vegetation (data S2 and Fig. 1) but retained these in our regression analyses as they were reflective of the surrounding grid cells.

**Table 2.** Taxa harmonized according to RPP (RPP taxa, *n* = 30) and grouped into land cover and plant functional types.

RPP taxa	Plant functional type	Land cover type
Amaranthaceae/Chenopodiaceae	Herbaceous plant Heathland shrubs	Open vegetation
*Artemisia*		
Cyperaceae		
*Filipendula*		
*Plantago lanceolata* type		
Poaceae		
*Rumex acetosa* type		
*Calluna vulgaris*		
Ericaceae		
*Juniperus*		
*Betula*	Shade-intolerant trees and shrubs Intermediate trees and shrubs*	Light woodland
*Pinus*		
*Pistacia*		
*Corylus*		
*Buxus sempervirens*		
*Phillyrea*		
*Quercus* deciduous		
*Taxus baccata*		
*Salix*		
*Abies*	Shade-tolerant trees	Closed forest
*Alnus*†		
*Carpinus betulus*		
*Carpinus orientalis/Ostrya carpinifolia*		
*Castanea sativa*		
*Fagus*		
*Fraxinus*†		
*Picea*		
*Quercus* evergreen‡		
*Tilia*		
*Ulmus*		

*Taxa that depend on open or semi-open conditions for the long-term maintenance of their populations.

†Taxa considered to be “shade-tolerant” due to their ability to thrive in shaded forest landscapes despite having high light requirements because of their tolerance to wet soils and/or ability to recruit in small treefall gaps.

‡*Quercus* evergreen is conservatively included as shade-tolerant, based on *Quercus ilex*. However, in the Mediterranean, other *Quercus* evergreen species may indicate more open conditions.

### Climate data and biomes

We used equilibrium simulations of the climate at 127 ka B.P. (the climatic optimum of the Last Interglacial) as in (*68*), from six Earth system models: AWI-ESM-1-1-LR (*69*), CNRM-CM6-1 (*70*), GISS-E2-1-G (*71*), INM-CM4-8 (*72*), IPSL-CM6-LR (*73*), and MIROC-ES2L (*74*). We downscaled the monthly surface air temperature and precipitation from these models to a resolution of 5 km. We then bias-corrected the values of the Last Interglacial simulation by comparing the historical simulations of the same models with the CHELSA V2 high-resolution climate dataset (*75*) over the period 1981–2010. From the corrected Last Interglacial values, we calculated the mean of the six models and derived bioclimatic variables, as in WorldClim (*76*).

We determined the climatic biomes of the Last Interglacial period using monthly temperature and precipitation data from each of the six Last Interglacial models and the mean ensemble model to produce the first Köppen-Geiger climate classification maps, as in (*77*), for the Last Interglacial period (fig. S7; Supplementary Materials). To maintain a large sample size, we grouped the Köppen-Geiger climate classifications into four main climate types for the analysis: oceanic, continental, subarctic, and Mediterranean (fig. S7 and table S3). We also included an Alpine category to separate this mountainous region from the predominantly lowland regions. The Alpine grid cells had continental or oceanic climates and experienced higher precipitation (>100 mm in the wettest month) and/or a higher SD of elevation (>500 m).

While we examined “*Quercus/Corylus*-dominated” and “*C. betulus*–dominated” vegetation in place of a given time window, the climate data reflected a specific time (127 ka). We acknowledge this limitation but argue that the interglacial climatic peak should correspond well to the temperate phase of vegetation (*24*), as well as to a more stable climate (*78*). Furthermore, macroclimatic variables exhibited broad trends. Although we cannot infer more localized events (*78*) from the available vegetation and climate data, elucidation of broader climatic trends is consistent with the aims of this study.

### Statistical analysis

#### Robustness assessment

We tested for a relationship between land-cover type and SE using a one-way analysis of variance (ANOVA) and Tukey’s post hoc test. We tested the reliability of the REVEALS model for data from the Last Interglacial period to establish its suitability for scarce data. Specifically, we examined grid cells with small basins that violated the assumptions of the REVEALS model (*27*). We compared the reconstructions using small lakes and bogs to those using large lakes to separate the effects of wetland vegetation at the margins of small lakes and bogs from those of regional grasslands (Supplementary Materials).

We used beta regression (Supplementary Materials) to test for a relationship between vegetation openness and bog presence as well as key wetland taxa, i.e., willow (*Salix*) and alder (*Alnus*). We also tested the relationship between lake size and vegetation openness. Because the REVEALS model relies on pollen deposited in large lakes, we wanted to ensure that any openness found was not an expansion of open woodlands near lake margins. We summed the radii of each lake per grid cell to form our explanatory variable and performed a beta regression analysis with vegetation openness as the response variable.

#### Drivers of vegetation openness

We used beta regression to test the relationship between vegetation openness and potential drivers thereof (explanatory variables) (*4*). These included precipitation and temperature extremes, degree of continentality (the difference between mean temperature of the warmest and coldest quarters) (*33*), latitude (to assess disequilibrium dynamics following glaciation) (*79*), and timing within an interglacial period. We included the SD of elevation to assess the role of terrain roughness on vegetation openness, for example, in relation to megaherbivore accessibility, where more energetically expensive sites (*49*) would be less grazed and therefore contain less open vegetation taxa (*15*). We also included a binary variable indicating whether a grid cell was Alpine or not, to capture any effect of lowland versus mountain areas (fig. S1). We used backward stepwise selection to exclude explanatory variables based on high-variance inflation factors (>5) and correlation coefficients (>0.2). Our final model contained six explanatory variables: mean temperature of the warmest quarter (°C), precipitation of the driest month (mm), degree of continentality (°C), SD of elevation (m), Alpine (0, 1), and time window (early-temperate and late-temperate).

For all analyses, we used R version 4.2.2 (2022-10-31). *P* value thresholds are given as graded measures of evidence, from “little or no evidence” to “very strong evidence” (*80*).

## Supplementary Materials

### This PDF file includes:

Supplementary Text Figs. S1 to S8 Tables S1 to S3 Legends for data S1 and S2 References
