# Supplementary material for: Substantial light woodland and open vegetation characterized the temperate forest biome before Homo sapiens
Source: Sci Adv. 2023 Nov 10;9(45):eadi9135. doi: 10.1126/sciadv.adi9135 (PMC10637746; doi:10.1126/sciadv.adi9135)
Supplement: Supplementary file 1 — Supplementary Text Figs. S1 to S8 Tables S1 to S3 Legends for data S1 and S2 References [file sciadv.adi9135_sm.pdf]

Science Advances
AAAS

Supplementary Materials for

Substantial light woodland and open vegetation characterized the temperate forest biome before *Homo sapiens*

Elena A. Pearce *et al.*

Corresponding author: Elena A. Pearce, elena.pearce@bio.au.dk

Sci. Adv. **9**, eadi9135 (2023)
DOI: 10.1126/sciadv.adi9135

The PDF file includes:

Supplementary Text
Figs. S1 to S8
Tables S1 to S3
Legends for data S1 and S2
References

Other Supplementary Material for this manuscript includes the following:

Data S1 and S2

Supplementary Text

REVEALS model

Pollen selection and harmonisation

For pollen selection and for running the REVEALS model, we used protocols developed by the LandClim and PAGES Land-cover6K projects (6, 7, 81) adapted to the Last Interglacial period. Relative pollen productivity (RPP) estimates exist for many European plant taxa, and there are several syntheses of RPP estimates and pollen fall speeds. Here, we used the most complete, up-to-date synthesis (7), which has been well-validated against modern data in Europe (62). We did not include *Secale* and Cerealia-type taxa as separate from Poaceae, given that the Last Interglacial occurred before the onset of agriculture. Where these taxa were present, we grouped them as wild plants under Poaceae. Additionally, in the absence of an RPP estimate for *Taxus baccata* (yew), an important taxon for the Last Interglacial, we used the estimate for *Juniperus* (juniper), as in Kuneš et al. (40). We excluded several entomophilous (insect-pollinated) taxa and rare taxa, as recent work has shown that the addition of a large number of such taxa does not improve REVEALS reconstructions (62, 63). We harmonised the taxonomy and nomenclature of pollen morphological types from the 96 pollen records, and then assigned them to 1 of 30 RPP-harmonised taxa (from now on referred to as RPP taxa; Table 2), following the protocol outlined in Githumbi et al. (7). The 30 RPP taxa were grouped to five plant functional types and three land cover types (Table 2), and aggregated into the Protocratic, early-Mesocratic (early-temperate), late-Mesocratic (late-temperate), and Telocratic zones. To check how representative the RPP taxa were of the whole pollen sample, we calculated the proportion of pollen grains within each sample that were included in the RPP taxa. The REVEALS taxa (n = 31) represented 95.5% ± 5.1 of the total terrestrial pollen sum.

Model Parameters

The REVEALS model relies on pollen sequences collected from large lakes. It has also been shown to work well with multiple smaller lakes or bogs, though the standard error is larger (63, 81). While it is therefore appropriate to use pollen records from small sites to increase the number of pollen records included in a REVEALS reconstruction, estimates using pollen assemblages from large bogs should be interpreted with caution (63; see Fig. S8). The REVEALS model requires estimates of basin size for sites used; however, the exact basin size can be difficult to define for interglacial deposits. Where possible, we obtained site type and radius information from original publications or directly from individual pollen data contributors. Otherwise, we measured the modern basin and/or elevation data following Mazier et al. (63). Finally, where this information was absent or uncertain, we assigned the basin size of remaining sites as small (5 m radius; n = 12).

We applied a Gaussian plume model for pollen dispersal and deposition models for bogs (29, 82) and lakes (27). We used a constant wind speed of 3 m s^{-1} (7, 81) and the maximum extent of the regional vegetation (Zmax) was set to 50 km, roughly corresponding to a 1°×1° grid cell (63).

Köppen-Geiger climate classification maps

When a palaeoclimate (i.e. the LIG) is simulated with different models, then differences in the setup of the models can produce different results because their sensitivities to a change in forcings is different, even though their present-day climates are similar (83). To know which model produced the best representation of the palaeoclimate, we used the distribution of *Ilex* (holly) as an indicator of oceanic climate (84), and compared this to the oceanic-continental boundary present in the classification maps. The eastern-most distribution of *Ilex* agreed with recent Maximum Likelihood Classification analysis (85) that an oceanic climate extended much further East in the Last Interglacial than today. We found that this pattern was best reflected in GISS-E2-1-G (71), and therefore used this model to produce our final climate classification map (Fig. S7) for regression analyses. For comparison, the mean ensemble model results are also presented in Fig. S7.

Robustness assessment: model comparisons

We produced REVEALS estimates for grid cells that included both reliable (model assumptions met: large lakes) and unreliable (model assumptions unmet: small lakes/bogs) sites (Fig. S8). For these grid cells, we ran REVEALS for 1) all reliable sites, 2) all unreliable sites, and 3) all reliable + unreliable sites (Fig. S6). We also wanted to test the suitability of using marine and riverine sites for REVEALS. These form only a small proportion (marine = 0.05; riverine = 0.03; Fig. S2) of our dataset, but also violate the assumptions of the REVEALS model. However, we did not have reliable sites within the same grid cells to use for comparison. We acknowledge this limitation in our dataset, and show the relevant, less reliable grid cells in Fig. S8.

Robustness assessment: local vegetation effects

In some grid cells, both Cyperaceae and Poaceae could represent wetland taxa, such as *Phragmites*, particularly in the early-temperate period and some Mediterranean grid cells (86). Floodplains are likely to characterise riverine sites (87), which explains the high proportions of herbaceous taxa found in all British Isles grid cells (Fig. 1). However, it is important to note that temperate floodplains in Europe today tend to become densely wooded in the absence of land use or restoration actions, whereas grasslands often dominated floodplains in the Last Interglacial period (6, 88). While the wetland vegetation surrounding the lakes and bogs likely had a small local effect in this study (7), validations of the REVEALS model against modern analogues in Europe (59, 60) and North America (61) (see Methods) showed that the model accounted well for the local effects of vegetation. Large bogs may present more uncertainty (32), but most of the pollen sequences used in this study were from lakes (Fig. S8). Furthermore, we found no effect for the presence of bogs or the wetland taxa *Alnus* and *Salix* on vegetation openness. We also found no correlation between lake size and vegetation openness, suggesting that the presence of open vegetation was not due to local expansion at lake margins. These results agree with the interpretations in the literature for the temperate forest biome during the Last Interglacial period. In Neumark Nord, Germany (Table S2), researchers found that high proportions of Poaceae and Cyperaceae did not represent increases in wetland-indicating taxa, but rather tracked increases in other non-arboreal pollen (20). Furthermore, the presence of dry grasslands, meadows, and other non-wetland open vegetation have been indicated by plant macrofossil, mollusc, and beetle

records (4, 8, 9); large herbivore diet analyses (34); and the presence of forb taxa that characterise grasslands and disturbed soils, such as *Artemisia,* Amaranthaceae/Chenopodiaceae, and *Rumex acetosa* (Data S1) (20). It is therefore likely that, for most of our grid cells in the temperate forest biome, the representation of open vegetation is reflective of regional cover in the Last Interglacial period and likely indicates grasslands.

Beta regression

We used beta regression using the "betareg" R package (89) to examine possible spatial, temporal and environmental drivers of vegetation openness. Beta regression is appropriate for modelling continuous proportion data (90) and produces two sub-models: a location model and a precision model (89). The location model predicts the mean and is estimated by a logit link. The precision model, with a log link, produces a *phi* coefficient, where the higher *phi* corresponds to higher precision or lower variance.

For the beta regression analyses, we removed an influencing outlier in Southern Norway. The reconstructed climatic data reported monthly precipitation for this grid cell at much greater values than expected – nearly twice the value than any other grid cell. We ran the model both with and without this grid cell and found that the results varied considerably. In the main text, we report results of the model that excluded the outlier, given our rationale that the precipitation values here are unreliable. Results including the outlier can be found in supplementary material (Fig. S3) for comparison.

Table S1. Mean REVEALS estimate and standard deviation (SD) per taxa per time window (early-temperate; late-temperate). Mean and SD measures are given for temperate lowland Europe (temperate forest biome, n = 45) and Europe – all (all grid cells, n = 66).

			**Open Vegetation**										**Light Woodland**								
Temperate Phase	Area	Measure	Amanranthaceae/ Chenopodiaceae	*Artemisia*	*Calluna vulgaris*	Cyperaceae	Ericaceae	*Filipendula*	*Juniperus*	*Plantago lanceolata* type	Poaceae	*Rumex acetosa* type	*Betula*	*Buxus sempervirens*	*Corylus avellana*	*Phillyrea*	*Pinus*	*Pistacia*	*Quercus* deciduous	*Salix*	*Taxus*
Early	Temperate Lowland Europe	Mean	0.07	0.22	0.62	6.41	0.1	0.02	0.08	0.02	10.18	0.09	3.01	0	35.95	0	8.11	0	8.9	0.43	0.79
Early	Temperate Lowland Europe	SD	0.1	0.28	1.01	9.19	0.33	0.05	0.41	0.1	13.83	0.17	6.12	0.03	18.66	0	6.57	0	5.93	0.57	1.26
Late	Temperate Lowland Europe	Mean	0.07	0.07	1.07	6.98	0.38	0.04	0.02	0.03	6.23	0.03	1.84	0.01	20.96	0	4.67	0	2.6	0.44	0.65
Late	Temperate Lowland Europe	SD	0.24	0.19	2.2	13.69	1.88	0.11	0.04	0.14	12.39	0.07	4.01	0.03	17.25	0	6.65	0	2.62	1.6	0.75
Early	Europe - all	Mean	0.08	0.36	0.42	7.3	0.13	0.02	0.14	0.03	10.69	0.09	2.43	0.01	30.08	0.03	7.01	0.05	8.55	0.37	1.09
Early	Europe - all	SD	0.14	1.23	0.88	13.61	0.37	0.04	0.42	0.11	16.18	0.18	5.48	0.04	21.5	0.17	7.89	0.23	6.73	0.54	1.86
Late	Europe - all	Mean	0.13	0.23	0.73	7.98	0.4	0.03	0.08	0.04	7.32	0.04	1.7	0.04	15.61	0.02	3.76	0.01	2.85	0.48	0.5
Late	Europe - all	SD	0.54	0.73	1.88	16.3	1.67	0.09	0.26	0.18	13.48	0.08	3.8	0.1	17.08	0.1	5.9	0.08	4.79	1.52	0.68

Temperate Phase	Area	Measure	*Abies alba*	*Alnus glutinosa*	*Carpinus betulus*	*Carpinus orientalis/ Ostrya carpinifolia*	*Castanea*	*Fagus sylvatica*	*Fraxinus*	*Picea*	*Quercus* evergreen	*Tilia*	*Ulmus*
			**Closed Forest**										
Early	Temperate Lowland Europe	Mean	0.52	1.42	3.45	0	0	0	2.22	2.98	0	4.7	5.27
Early	Temperate Lowland Europe	SD	1.61	1.64	7.19	0	0	0.01	1.77	5.64	0	5.3	4.21
Late	Temperate Lowland Europe	Mean	7.98	1.93	19.61	0	0	0	1.03	6.66	0	4.38	3.42
Late	Temperate Lowland Europe	SD	13.85	1.66	15.99	0	0	0.01	0.94	8.09	0	4.62	3.4
Early	Europe - all	Mean	6.34	1.05	2.55	0.65	0	0.14	2.32	3.56	0.09	3.39	4.93
Early	Europe - all	SD	18.07	1.47	6.11	4.19	0	0.66	2.28	6.84	0.67	4.78	4.21
Late	Europe - all	Mean	18.02	1.4	14.45	0.62	0	0.1	0.86	5.61	0.1	3.19	3.11
Late	Europe - all	SD	28.91	1.59	15.39	4.68	0	0.6	0.96	7.35	0.78	4.38	5.31

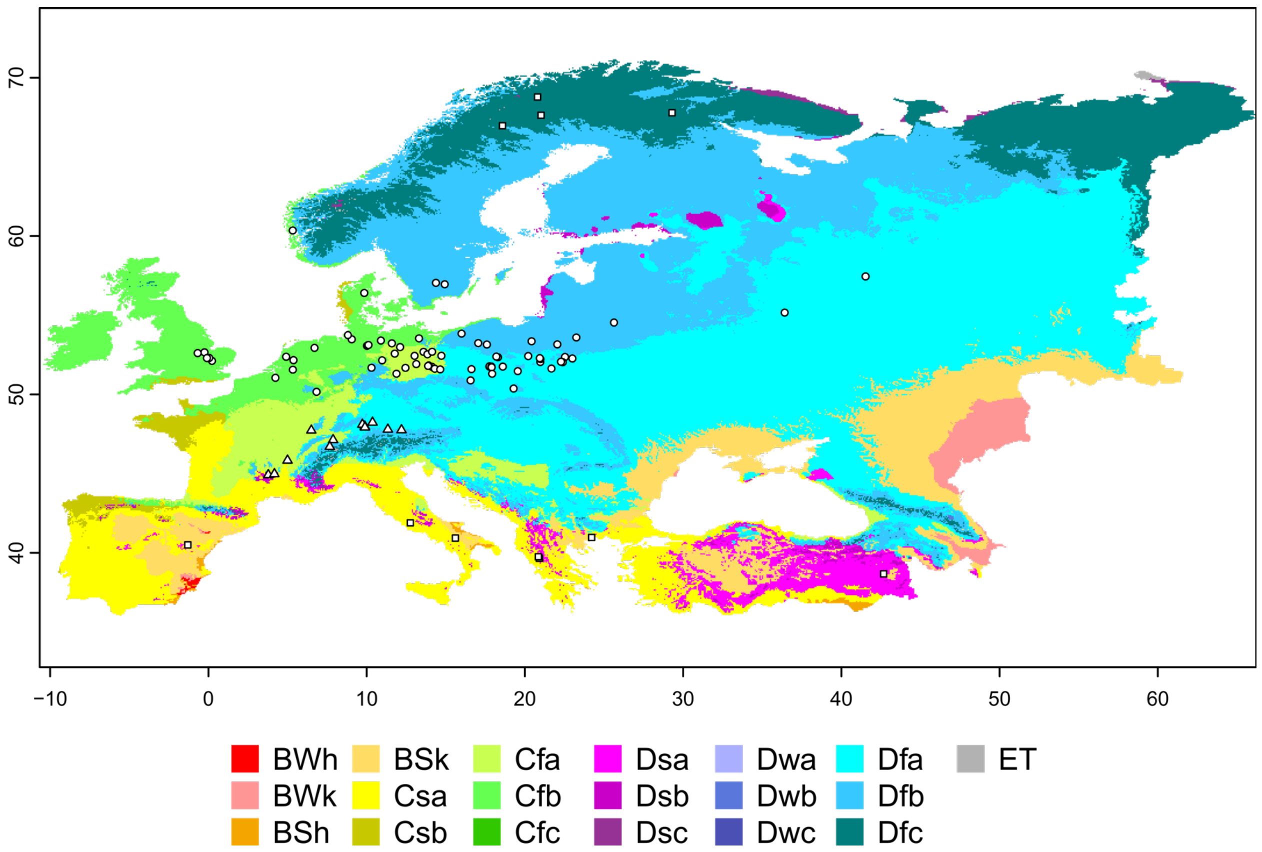

Fig. S1. Location of 96 pollen sequences (points; see Table S2) divided according to Köppen-Geiger climate classification for the Last Interglacial. We reconstructed the classifications as in Beck et al. (77), from monthly temperature and precipitation data from the GISS-E2-1-G earth system model (71). For further methods and choice of model, see "climate data and zones" in main methods. Circular points are within the temperate forest biome, triangular points show "Alpine" sites, and square points the remaining sites (Mediterranean and sub-Arctic).

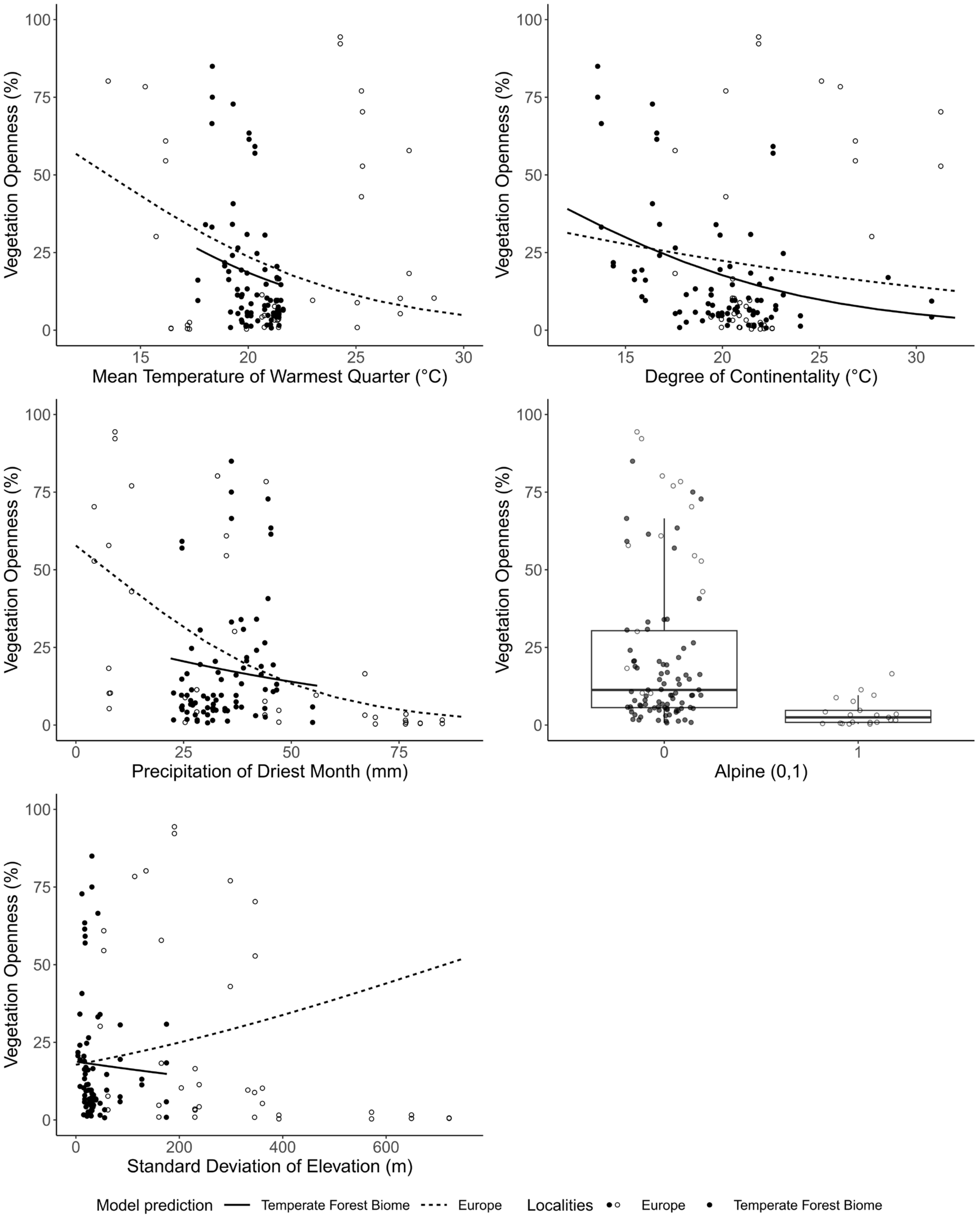

Fig. S2. Full model results of beta regression analysis, with the influencing outlier in Southern Norway (grid cell 1021) excluded.

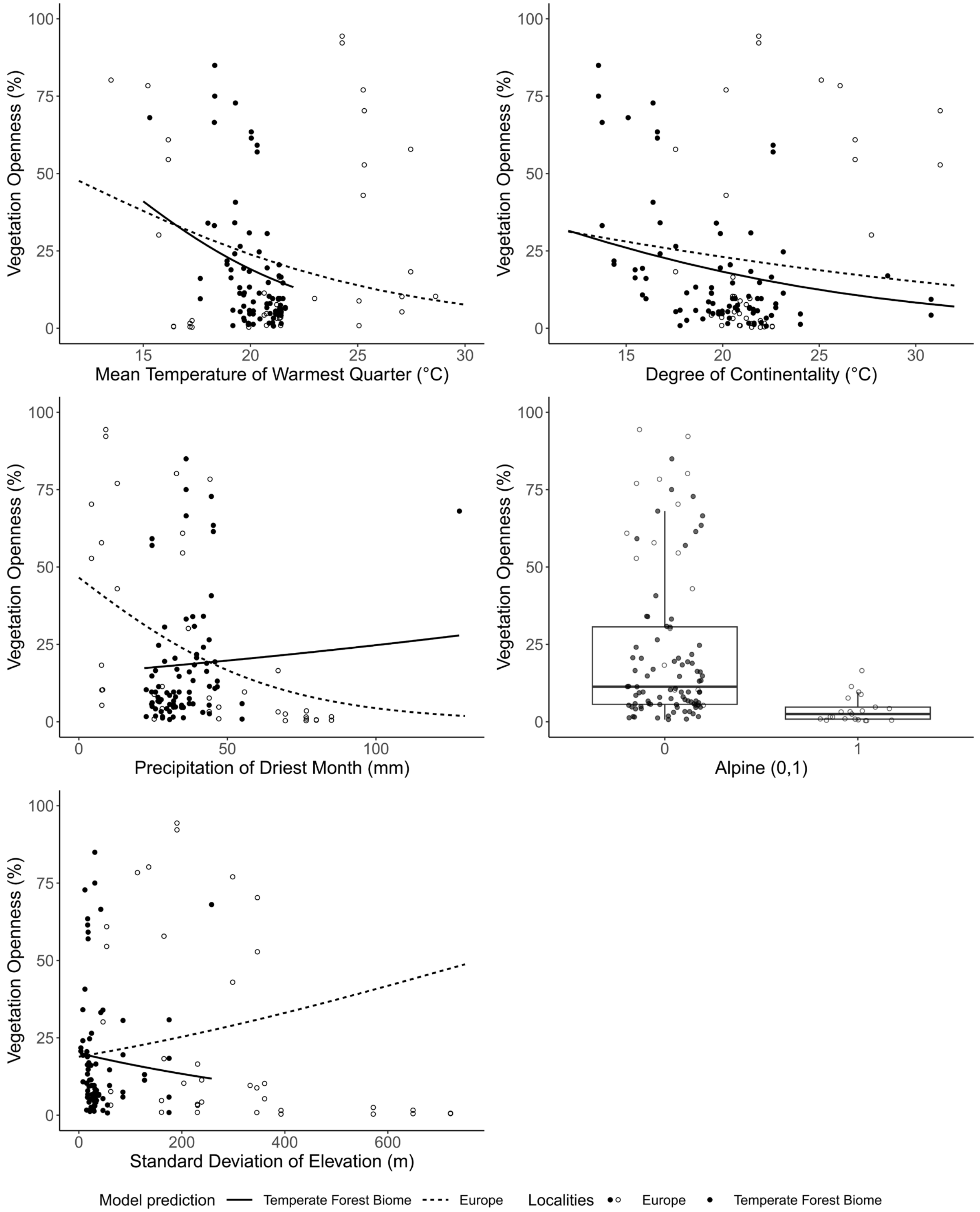

Fig. S3. Full model results of beta regression analysis, with the influencing outlier in South Norway (grid cell 1021) included.

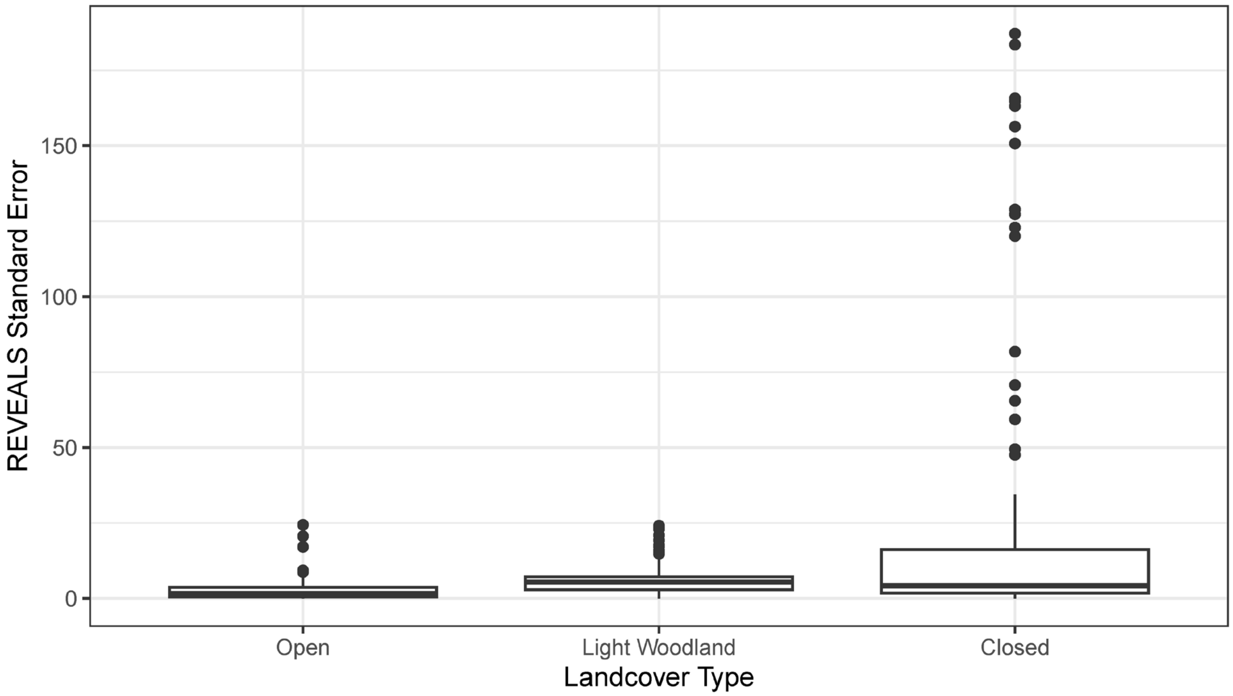

Fig. S4. Standard Errors from the REVEALS model, for all grid cells across all time windows, plotted by land cover type.

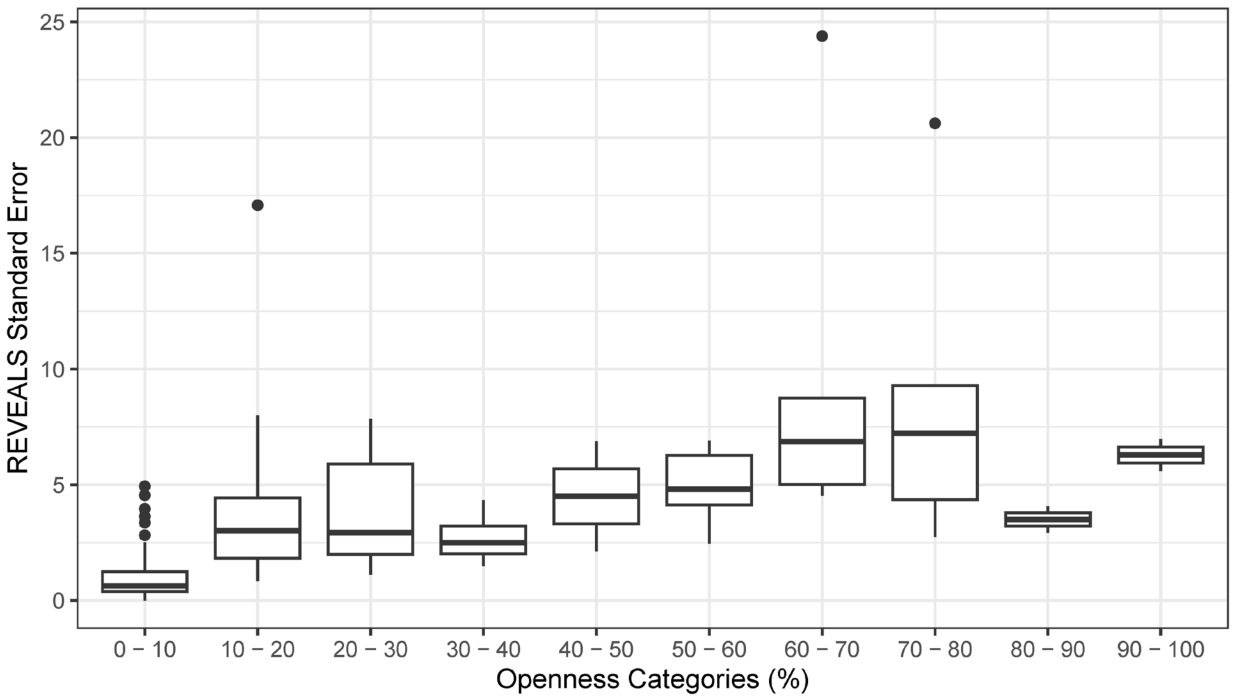

Fig. S5. Standard Errors from the REVEALS model, for all grid cells across all time windows, plotted by openness categories (%).

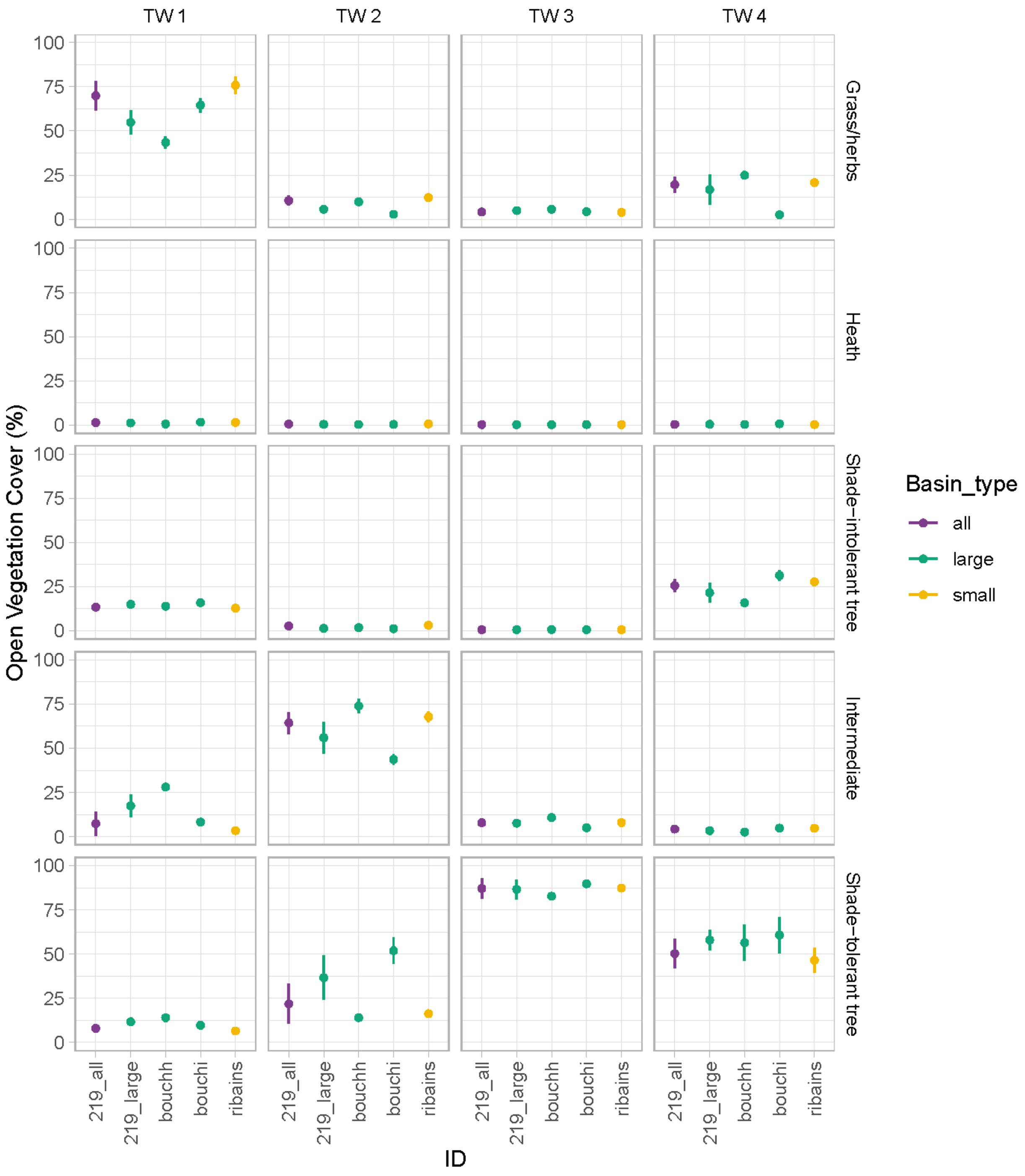

TW 1
TW 2
TW 3
TW 4
Grass/herbs
Heath
Shade−intolerant tree
Intermediate
Shade−tolerant tree
Open Vegetation Cover (%)
100
75
50
25
0
219_all
219_large
bouchh
bouchi
ribains
ID
Basin_type
all
large
small

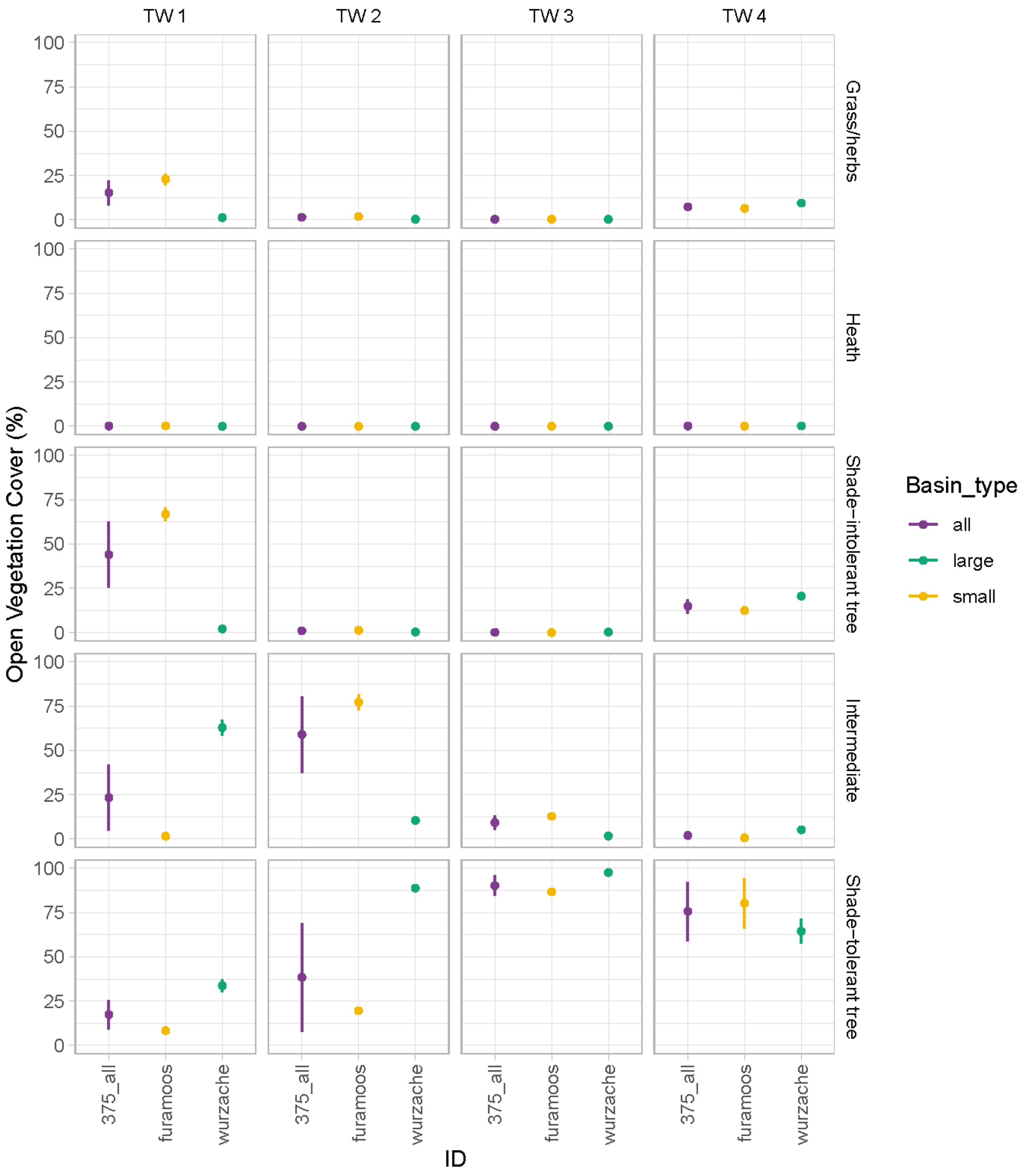
TW 1
TW 2
TW 3
TW 4
Grass/herbs
Heath
Shade−intolerant tree
Intermediate
Shade−tolerant tree
Open Vegetation Cover (%)
100
75
50
25
0
375_all
furamoos
wurzache
ID
Basin_type
all
large
small

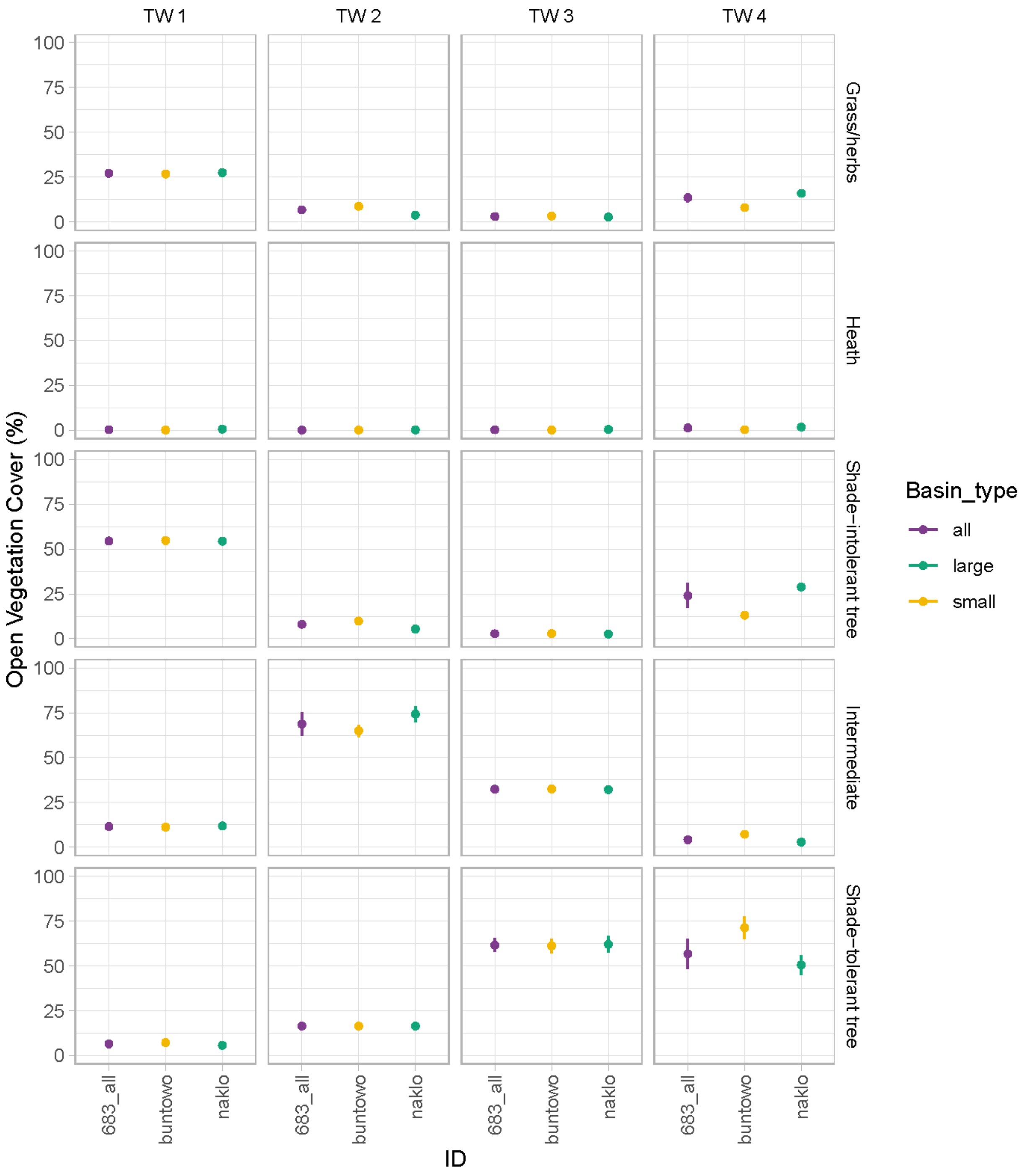
TW 1
TW 2
TW 3
TW 4
Grass/herbs
Heath
Shade−intolerant tree
Intermediate
Shade−tolerant tree
Open Vegetation Cover (%)
100
75
50
25
0
683_all
buntowo
naklo
ID
Basin_type
all
large
small

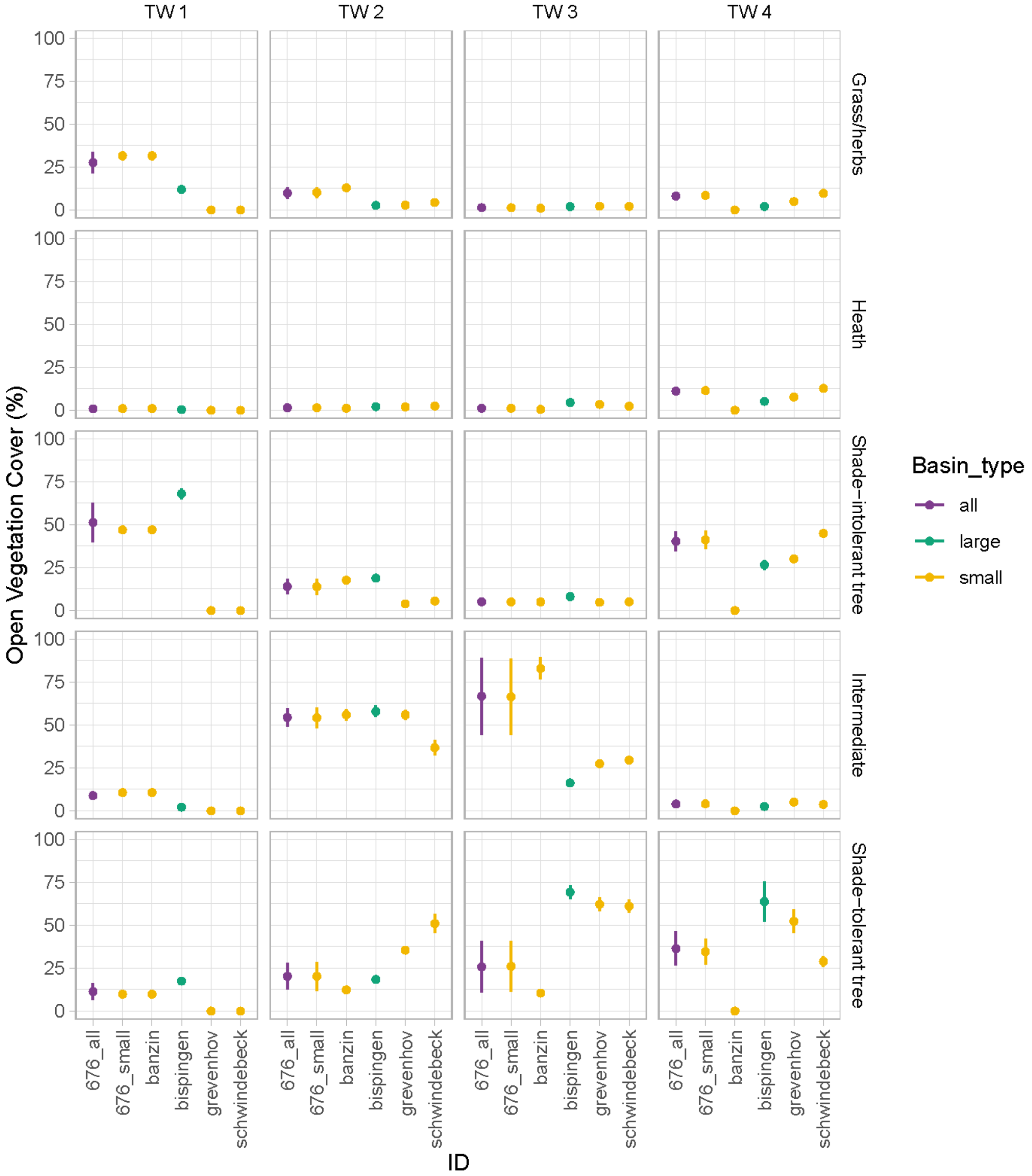

Fig. S6. Grid cell-based REVEALS estimates of vegetation openness (left Y-axis) for each PFT (right Y-axis) in grid cells 219, 375, 676, and 683 (Fig. S1, Table S2). Four time windows (top X-axis – TW1, TW2, TW3 and TW4: Protocratic, early-temperate (early Mesocratic), late-temperate (late Mesocratic) and Oligo/Telocratic, respectively) are shown. Pollen assemblages from 1-3 small lakes/bogs (yellow circles; <400 m radius) and 1-3 large lakes (green circles; 400 - 1300 m radius) were used for the REVEALS reconstructions for each time window. The REVEALS estimates obtained with pollen data from the single lakes/bogs, and the REVEALS

estimates obtained with pollen data from all lakes/bogs (purple circles), are shown with standard errors. We also grouped lakes/bogs according to size (676_small and 219_large) for further comparison to single large or small sites, respectively. Where standard error is very small, the error bars are smaller than the symbol size and therefore not visible.

Table S2. Metadata for all pollen sequences.

Pollen Data Contributor	Pollen Sequence	Sequence Country	Database	Lon (°)	Lat (°)	Radius (m)	Model	Publication(s)	DOI
Steve Boreham	Abington Hall G	United Kingdom	Authors	0.23	52.1	550	Lake	Boreham & Leszczynska (91)	https://doi.org/10.3390/quat2030024
Jeroen Schokker	Amersfoort I (B32B0119)	Netherlands	Geological Survey of the Netherlands	5.38	52.2	1500	Lake	Zagwijn, (92); Cleveringa et al. (93)	NA
Werner Ricken	Aschenhütte	Germany	Pangaea	10.3	51.7	43	Lake	Ricken & Grüger, (94)	https://doi.pangaea.de/10.1594/PANGAEA.729508
Jeroen Schokker	Amsterdam-Terminal (B25E0913)	Netherlands	Geological Survey of the Netherlands	4.91	52.4	10000	Lake	Van Leeuwen et al. (95)	https://doi.org/10.1017/S0016774600023647
Jeroen Schokker	Amersfoort New (B32B2092)	Netherlands	Geological Survey of the Netherlands	5.38	52.2	1500	Lake	Kasse et al. (96)	https://doi.org/10.1017/NJG.2022.4
Anna Hrynowiecka	Banzin	Germany	Authors	10.9	53.4	5	Lake	Börner et al. (97)	https://doi.org/10.1016/j.quaint.2014.10.022
Anna Hrynowiecka	Beckenthin	Germany	Authors	11.6	53.2	30	Bog	Hrynowiecka et al. (98)	https://doi.org/10.1016/j.quaint.2021.01.025
Małgorzata Malkiewicz	Bieganin	Poland	Authors	17.8	51.8	110	Lake	Malkiewicz (99)	NA
Helmut Müller	Bispingen	Germany	Neotoma	10	53.1	564	Lake	Müller (100)	doi:10.21233/gx58-4a75
Jaqueline Strahl	Borgisdorf	Germany	Geological Survey of Brandenburg	13.1	51.9	5	Lake	Hermsdorf & Strahl (101)	NA
Jeroen Schokker	Boxtel-Breede Heide 2 (B51B0307)	Netherlands	Authors	5.34	51.6	100	Bog	Schokker, Cleveringa & Murray (102)	https://doi.org/10.1002/jqs.808
Malgorzata Malkiewicz	Buntowo	Poland	EECRG(digitised)	17.1	53.2	356	Lake	Malkiewicz (103)	https://doi.org/10.1016/j.quaint.2016.07.006
Olga K. Borisova	Butovka	Russia	EPD	36.4	55.2	219	Bog	Borisova (104)	NA
Krzysztof Bińka	Czaple	Poland	Authors	22.5	52.4	75	Lake	Bińka & Nitychoruk (105)	https://doi.org/10.1016/j.revpalbo.2010.11.003
Tim Mighall	Deeping St James	United Kingdom	Authors	-0.25	52.7	22.2	Lake	Keen et al. (106)	https://doi.org/10.1002/(SICI)1099-1417(199908)14:5%3C411::AID-JQS447%3E3.0.CO;2-M
Wilhelmus De Gans	Drentsche Valley	Netherlands	Digitised	52.9	6.71	24	Lake	De Gans (107)	NA
Krzysztof Bińka	Dziewule	Poland	Authors	22.4	52	60	Lake	Bińka & Nitychoruk (108)	NA
Jaqueline Strahl	Eichow	Germany	Geological Survey of Brandenburg	14.1	51.7	166.41	Lake	Hermsdorf & Strahl (101)	NA
Hans-Jürgen Beug	Eurach	Germany	Pangaea	11.3	47.8	4184.1	Lake	Beug (109)	https://doi.org/10.1594/PANGAEA.58939
Jan Mangerud	Fjøsanger	Norway	EECRG(digitised)	5.33	60.4	1204.8	Lake	Mangerud, Sejrup, Sønstegaard & Haldorsen (110)	https://doi.org/10.1111/j.1502-3885.1981.tb00479.x
Ulrich C. Müller	Füramoos	Germany	EPD	9.88	48	138.2	Lake	Müller, Pross & Bibus (111)	doi:10.1016/S0033-5894(03)00005-X
Jaqueline Strahl	Gloewen	Germany	Geological Survey of Brandenburg	12.1	53	5	Lake	Hermsdorf & Strahl (101)	NA
Jerzy Niklewski	Glówczyn G2	Poland	Pangaea	20.2	52.4	143.84	Lake	Niklewski (112)	https://doi.pangaea.de/10.1594/PANGAEA.739263
Zofia Janczyk-Kopikowa	Golkow	Poland	Pangaea	21	52.1	195.44	Lake	Janczyk-Kopikowa (113)	https://doi.org/10.1594/PANGAEA.760378
Samuel Wegmüller	Gondiswil-Seilern	Switzerland	EECRG(digitised)	7.88	47.1	79.788	Bog	Wegmüller (114)	NA

Karl-Ernst Behre	Grevenhof	Germany	Pangaea	10.1	53.1	127.41	Lake	Behre (115)	https://doi.pangaea.de/10.1594/PANGAEA.894223
Lothar Eissmann	Grobern94	Germany	EPD	12.5	51.7	1082.3	Lake	Eissman & Litt (116)	NA
Małgorzata Malkiewicz	Grudzielec	Poland	Authors	17.8	51.8	5	Lake	Malkiewicz (117)	doi:10.5586/asbp.2002.037
Małgorzata Malkiewicz	Gutów	Poland	Authors	17.9	51.8	15	Lake	Malkiewicz (118)	
Jaqueline Strahl	Hinterste Mühle	Germany	Geological Survey of Brandenburg	13.3	53.5	10	Lake	Strahl (119)	NA
Svend Th. Andersen	Hollerup	Denmark	Neotoma	9.85	56.4	150	Lake	Andersen (120)	doi:10.21233/z3x6-1z53
Wojciech Granoszewski	Horoszki Duże	Poland	Polish Pleistocene Pollen database	23	52.3	5	Lake	Granoszewski (121)	NA
Kaziemira Mamakowa	Imbramowice	Poland	EPD	16.6	50.9	282.09	Lake	Mamakowa (122)	NA
Polychronis C. Tzedakis	Ioannina 249	Greece	EPD	20.9	39.7	269.4	Lake	Tzedakis, Frogley & Heaton (123)	https://doi.org/10.1016/S0921-8181(02)00182-0
Polychronis C. Tzedakis	Ioannina 284	Greece	Authors	20.9	39.8	269.4	Lake	Tzedakis, Frogley & Heaton (123)	https://doi.org/10.1016/S0921-8181(02)00182-0
Bożena Noryśkiewicz	Jałówka	Poland	Authors	23.2	53.6	100	Lake	Rychel et al. (124)	https://doi.org/10.1016/j.quaint.2013.09.018
Ulrich C. Müller	Jammertal	Germany	Neotoma	9.73	48.1	356.82	Bog	Müller (125)	doi:10.21233/5pjf-4p55
Ann-Marie Robertsson	Ketkijärvet	Sweden	Authors	20.8	68.8	995	Lake	Robertsson & Rodhe (126)	https://doi.org/10.1111/j.1502-3885.1988.tb00564.x
Jaqueline Strahl	Kittlitz	Germany	Geological Survey of Brandenburg	13.9	51.8	25	Lake	Erd (127)	NA
Zofia Janczyk-Kopikowa	Kletnia Stara	Poland	Pangaea	21.7	51.6	126.16	Lake	Żarski (128)	https://doi.pangaea.de/10.1594/PANGAEA.739381
Burkhard Frenzel	Krumbach I	Germany	Pangaea	10.4	48.2	218.51	Lake	Frenzel (129)	https://doi.pangaea.de/10.1594/PANGAEA.736436
Bożena Noryśkiewicz	Kwiatków Las	Poland	Authors	17.9	51.7	15	Lake	Noryśkiewicz (130)	NA
Jacques-Louis de Beaulieu, Maurice Reille	La Grande Pile	France	EPD	6.5	47.7	282.09	Bog	Kukla et al. (131)	https://doi.org/10.1006/qres.2002.2340
Jacques-Louis de Beaulieu, Maurice Reille	Lac du Bouchet H	France	EPD	3.78	44.9	635.81	Lake	Reille et al. (132)	https://doi.org/10.1016/S0277-3791(97)00093-0.
Jacques-Louis de Beaulieu, Maurice Reille	Lac du Bouchet I	France	EPD	3.78	44.9	635.81	Lake	Reille et al. (132)	https://doi.org/10.1016/S0277-3791(97)00093-0
Jaqueline Strahl	Ladeburg	Germany	Geological Survey of Brandenburg	13.6	52.7	5	Lake	Hermsdorf & Strahl, (101)	NA
Nadine Pickarski	Lake Van	Turkey	Pangaea	42.7	38.7	33729	Lake	Pickarski (133)	https://doi.pangaea.de/10.1594/PANGAEA.853729
Zofia Balwierz	Łanięta	Poland	Authors	19.3	50.4	800	Lake	Balwierz & Roman (134)	NA
Małgorzata Malkiewicz	Lechitów	Poland	Authors	16.6	51.6	5	Lake	Malkiewicz (117)	doi:10.5586/asbp.2002.037
Jacques-Louis de Beaulieu, Maurice Reille	Les Echets	France	Authors	5	45.8	2034.2	Bog	de Beaulieu & Reille (65)	https://doi.org/10.1111/j.1502-3885.1984.tb00066.x

Jan Lundqvist	Leveäniemi	Sweden	EECRG(digitised)	21	67.6	406.84	Lake	Lundqvist (135)	NA
Krzysztof M Krupinski	Lomzyca Lomza2	Poland	Pangaea	22.1	53.2	472.03	Lake	Krupinski (136)	https://doi.pangaea.de/10.1594/PANGAEA.711922
Steve Boreham, Cunhai Gao	Mannings Farm Beds	United Kingdom	Authors	0.05	52.3	20	Lake	Gao & Boreham (87)	https://doi.org/10.1111/j.1502-3885.2010.00191.x
Vaida Šeirienė	Medininkai117	Lithuania	Authors	25.6	54.5	30	Lake	Šeirienė, Kühl & Kisielienė (137)	https://doi.org/10.1016/j.yqres.2014.04.004
Małgorzata Nita	Mikorzyn I Mlodszy	Poland	Authors	18.3	52.4	225	Lake	Stankowski & Nita (138)	NA
Judy Allen	Monticchio	Italy	Pangaea	15.6	40.9	3612.6	Lake	Allen et al. (139)	https://doi.org/10.1594/PANGAEA.707088
Bożena Noryśkiewicz	Nakło	Poland	Pangaea	17.6	53.2	437.02	Lake	Noryśkiewicz (140)	https://doi.pangaea.de/10.1594/PANGAEA.739576
Jaqueline Strahl	Nedlitz	Germany	Geological Survey of Brandenburg	13	52.4	5	Lake	Hermsdorf & Strahl (101)	NA
Corrie Bakels	Neumark Nord	Germany	Authors	11.9	51.3	1723	Lake	Bakels (141)	https://doi.org/10.1016/j.revpalbo.2012.06.003
Krzysztof Bińka	Nidzica	Poland	Authors	20.4	53.4	750	Lake	Bińka, Nitychoruk & Dzierżek (142)	https://doi.org/10.1111/j.1502-3885.2010.00179.x
Anna Brostrom, Per Möller	Nybygget	Sweden	Authors	14.9	57	2154.1	Lake	Lemdahl et al. (143)	https://doi.org/10.1002/jqs.2664
Karl-Ernst Behre	Oerel 61	Germany	EPD	9.06	53.5	250	Lake	Behre & van der Plicht (144)	https://doi.org/10.1007/BF00206091
Karl-Ernst Behre	Osterwanna	Germany	EPD	8.82	53.7	23.937	Bog	Behre, Göttlich & Werner (145)	NA
Hanna Winter	Ostrow	Poland	Pangaea	19.6	51.5	35.682	Lake	Klatkowa & Winter (146)	https://doi.pangaea.de/10.1594/PANGAEA.739625
Olga K. Borisova	PLES	Russia	EPD	41.5	57.5	500	Lake	Borisova (147)	https://doi.org/10.1016/j.quascirev.2007.07.001
Hanna Winter	Radówek	Poland	Authors	14.7	52.4	218.51	Bog	Urbański & Winter (148)	NA
Jaqueline Strahl	Rehnsdorf	Germany	Geological Survey of Brandenburg	14.3	51.6	60	Lake	Kühner & Strahl, (149)	doi:10.1127/1860-1804/2008/0159-0191
Jacques-Louis de Beaulieu	Ribains maar	France	EPD	3.78	44.9	141.61	Bog	de Beaulieu & Reille (150)	https://doi.org/10.1007/BF00189500
Hanna Winter	Rzecino	Poland	Authors	16	53.8	400	Lake	Winter, Dobracka & Ciszek (151)	NA
Eberhard Grüger	Samerberg I	Germany	Pangaea	12.2	47.8	100	Lake	Grüger (152)	https://doi.pangaea.de/10.1594/PANGAEA.726866
Jaqueline Strahl	Schönfeld	Germany	Geological Survey of Brandenburg	13.9	51.8	115	Lake	Erd (153)	NA
Brigitte Urban	Schöningen	Germany	Authors/Neotoma	11	52.2	500	Lake	Urban et al (154)	https://doi.org/10.3285/eg.41.1.07
Karl-Ernst Behre	Schwindebeck	Germany	Pangaea	10.1	53.1	39.894	Lake	Behre (155)	https://doi.pangaea.de/10.1594/PANGAEA.894473
Ann-Marie Robertsson	Seitevare	Sweden	Authors	18.6	67	12500	Lake	Robertsson & Rodhe (126)	https://doi.org/10.1111/j.1502-3885.1988.tb00564.x
Filip Van Beirendonck	Sint-Amands	Belgium	Authors	4.23	51	750	Bog	Verbruggen (156)	https://doi.org/10.20341/gb.2014.013
Małgorzata Nita	Slawoszewek 1999	Poland	Polish Pleistocene Pollen database	18.2	52.4	62.5	Lake	Stankowski, Bluszcz & Nita (157)	NA
J. Sakari Salonen	Sokli	Finland	Authors	29.3	67.8	2500	Lake	Salonen et al. (158)	https://doi.org/10.1038/s41467-018-05314-1

Patrick Schläfli	Spiezberg	Switzerland	Authors	7.67	46.7	8500	Lake	Schläfli et al. (159)	https://doi.org/10.1016/j.quascirev.2021.106975
Valérie Andrieu-Ponel	St. Front	France	Authors	4.17	45	309.02	Lake	Martin et al. (160)	https://doi.org/10.1016/j.orggeochem.2019.06.005
Anna Brostrom, Per Möller	Stora Gäddevik	Sweden	Authors	14.4	57	2417.2	Lake	Lemdahl et al. (143)	https://doi.org/10.1002/jqs.2664
Jaqueline Strahl	Strausberg	Germany	Geological Survey of Brandenburg	13.8	52.5	5	Lake	Hermsdorf & Strahl (101)	NA
Małgorzata Malkiewicz	Szklarka	Poland	Authors	17.9	51.3	250	Lake	Malkiewicz (161)	https://doi.org/10.1016/j.quaint.2016.09.026
Alice M. Milner	Tenaghi Philippon 2005	Greece	Authors	24.2	41	9339	Bog	Milner et al. (162)	https://doi.org/10.1016/j.quascirev.2016.10.016
Frank Sirocko	Trockenmaar _HL2	Germany	Pangaea	6.84	50.2	282.09	Lake	Sirocko et al. (163)	https://doi.pangaea.de/10.1594/PANGAEA.472208
Jaqueline Strahl	Tschernitz	Germany	Geological Survey of Brandenburg	14.6	51.6	5	Lake	Hermsdorf & Strahl (101)	NA
Jaqueline Strahl	Uchte flats	Germany	Geological Survey of Brandenburg	11.8	52.6	85	Lake	NA	NA
Piotr Kołaczek	Ustków	Poland	Authors	18.6	51.8	50	Lake	Kołaczek et al. (164)	https://doi.org/10.1016/j.quaint.2012.05.004
Donatella Magri	Valle Di Castiglione	Italy	Authors	12.8	41.9	500	Lake	Follieri, Magri, & Sadori (165)	https://doi.org/10.1016/1040-6182(89)90076-1
Jaqueline Strahl	Vevais	Germany	Geological Survey of Brandenburg	14.1	52.7	30	Lake	Lüthgens et al. (166)	https://doi.org/10.1016/j.quaint.2010.06.026
Penélope González-Sampériz	Villarquemado	Spain	Authors	-1.3	40.5	1828.2	Lake	González-Sampériz et al. (86)	https://doi.org/10.1016/j.quascirev.2020.106425
Krzysztof M Krupinski	Warszawa Kasprzak St	Poland	Pangaea	21	52.2	375	Lake	Krupinski & Morawski (167)	https://doi.pangaea.de/10.1594/PANGAEA.739950
Krzysztof M Krupinski	Warszawa Wawrzyszew 15	Poland	Pangaea	20.9	52.3	398.94	Lake	Krupinski & Morawski (167)	https://doi.pangaea.de/10.1594/PANGAEA.739948
Allan Hall	Wing	UK	EECRG(digitised)	-0.68	52.6	50	Lake	Hall (168)	https://doi.org/10.1111/j.1469-8137.1978.tb01655.x
Irena Agnieszka Pidek	Wiśniew II	Poland	Authors	22.3	52.1	5	Lake	Pidek & Terpiłowski (169)	NA
Steve Boreham	Woolpack Farm site	United Kingdom	Authors	-0.1	52.3	15	Lake	Gao et al. (48)	https://doi.org/10.1016/S0277-3791(99)00028-1
Eberhard Grüger	Wurzach	Germany	Pangaea	9.89	47.9	500	Lake	Grüger & Schreiner (170)	https://doi.pangaea.de/10.1594/PANGAEA.713702

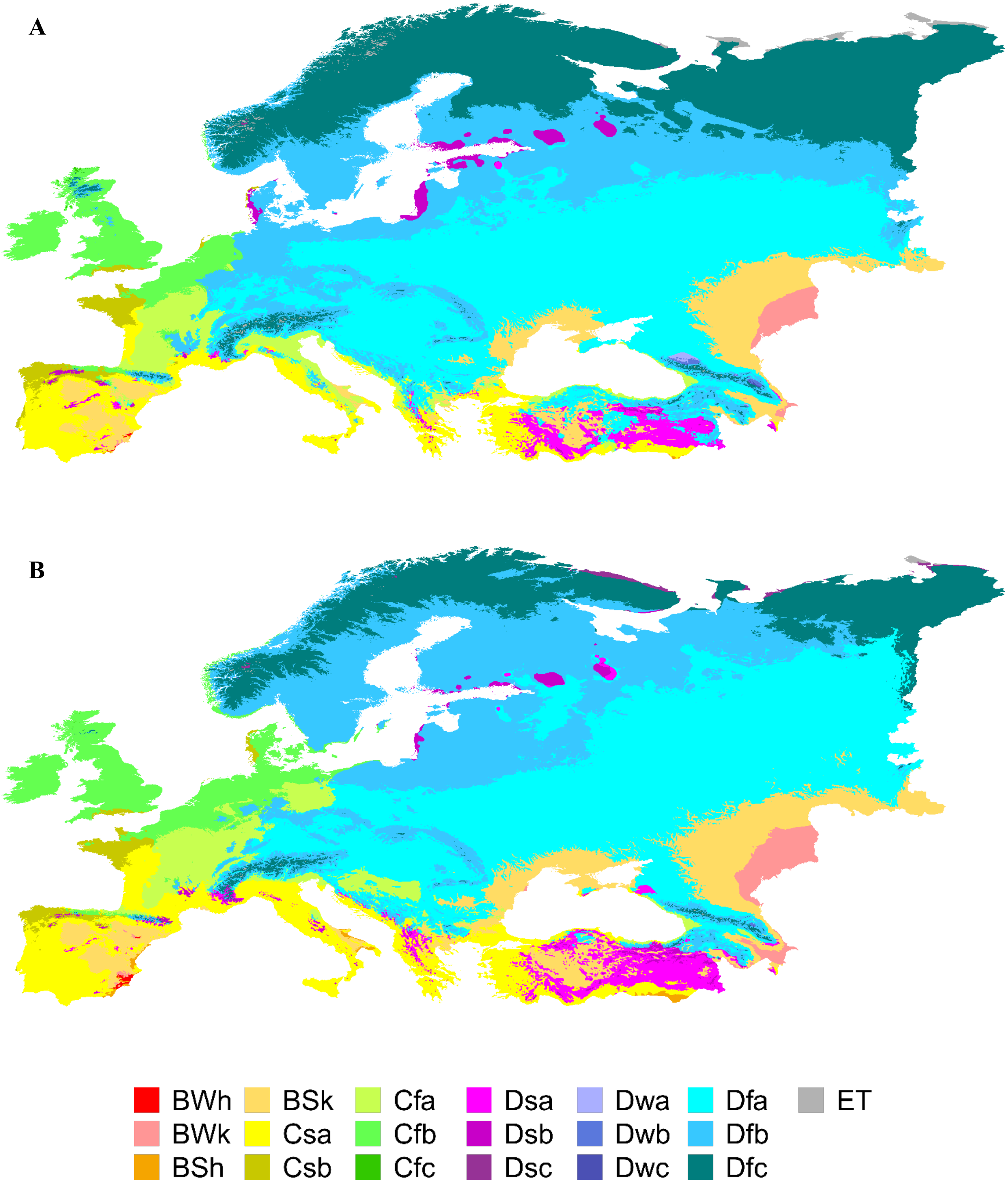

Fig. S7. Köppen-Geiger climate classification maps produced with mean ensemble model (A) and GISS-E2-1-G model (B; 71). The colour scheme was adopted from Beck et al. (77).

Table S3: Main climate zones covered by the 96 pollen sequences, grouped according to their description in the paper.

Dfc	Cold, no dry season, cold summer	Subarctic
Dfb	Cold, no dry season, warm summer	Continental
Dfa	Cold, no dry season, hot summer	
Cfb	Temperate, no dry season, warm summer	Oceanic
Cfa	Temperate, no dry season, hot summer	
Csa	Temperate, dry season, hot summer	Mediterranean
BSk	Arid, steppe, cold	
Dsa	Cold, dry season, hot summer	

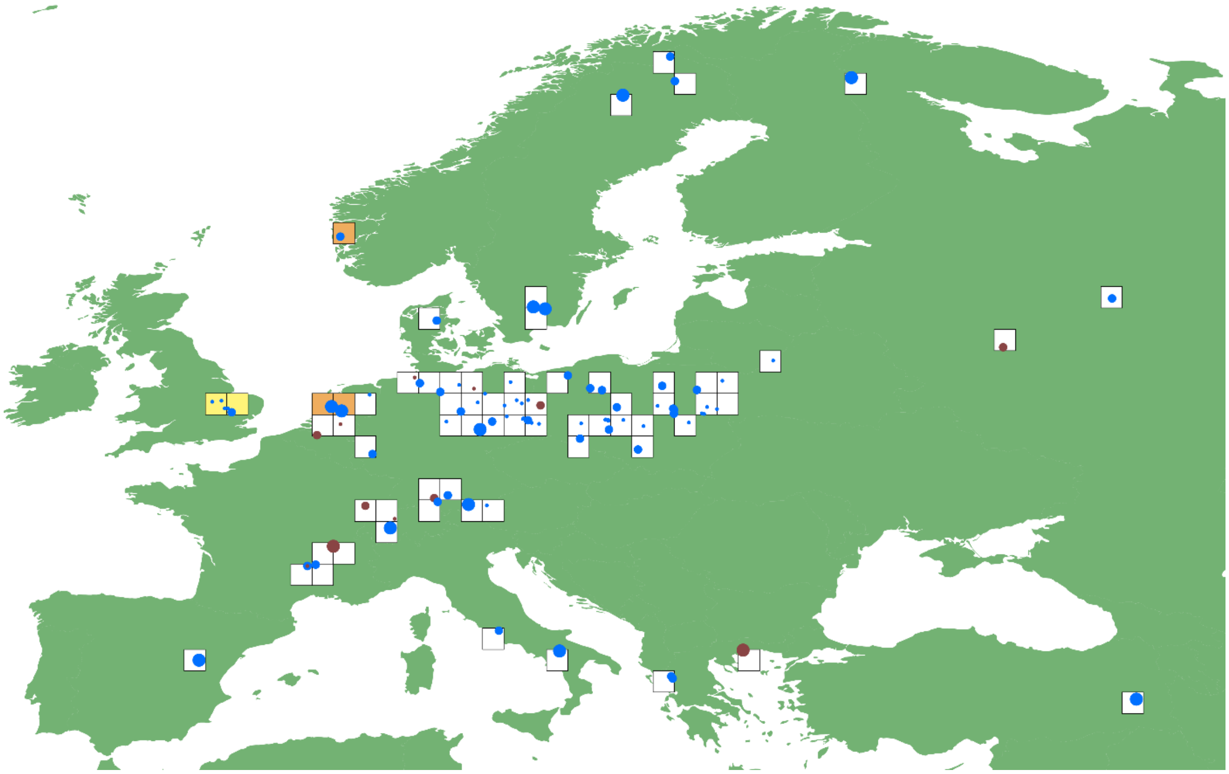

Fig. S8. Map of individual pollen sequences taken from lakes (blue points) and bogs (brown points). Small sites (<150 m radius), medium sites (150 – 1500 m radius) and large sites (>1500 m radius) correspond to small, medium and large points. Riverine (yellow) and marine (orange) grid cells are considered less reliable.

Data S1. (separate file)
REVEALS mean percentage cover estimates for each RPP taxon per grid cell.

Data S2. (separate file)
REVEALS mean standard error estimates for each RPP taxon per grid cell.

REFERENCES AND NOTES

1. G. F. Peterken, *Natural Woodland: Ecology and Conservation in Northern Temperate Regions* (Cambridge University Press, 1996).

2. F. J. G. Mitchell, How open were European primeval forests? Hypothesis testing using palaeoecological data. *J. Ecol.* **93**, 168–177 (2005).

3. F. W. M. Vera, *Grazing Ecology and Forest History* (CABI Publishing, 2000).

4. J.-C. Svenning, A review of natural vegetation openness in north-western Europe. *Biol. Conserv.* **104**, 133–148 (2002).

5. A. Feurdean, E. Ruprecht, Z. Molnár, S. M. Hutchinson, T. Hickler, Biodiversity-rich European grasslands: Ancient, forgotten ecosystems. *Biol. Conserv.* **228**, 224–232 (2018).

6. M.-J. Gaillard, S. Sugita, F. Mazier, A.-K. Trondman, A. Broström, T. Hickler, J. O. Kaplan, E. Kjellström, U. Kokfelt, P. Kuneš, C. Lemmen, P. Miller, J. Olofsson, A. Poska, M. Rundgren, B. Smith, G. Strandberg, R. Fyfe, A. B. Nielsen, T. Alenius, L. Balakauskas, L. Barnekow, H. J. B. Birks, A. Bjune, L. Björkman, T. Giesecke, K. Hjelle, L. Kalnina, M. Kangur, W. O. van der Knaap, T. Koff, P. Lagerås, M. Latałowa, M. Leydet, J. Lechterbeck, M. Lindbladh, B. Odgaard, S. Peglar, U. Segerström, H. von Stedingk, H. Seppä, Holocene land-cover reconstructions for studies on land cover-climate feedbacks. *Clim. Past.* **6**, 483–499 (2010).

7. E. Githumbi, R. Fyfe, M. Gaillard, A. Trondman, F. Mazier, A. Nielsen, A. Poska, S. Sugita, J. Woodbridge, J. Azuara, A. Feurdean, R. Grindean, V. Lebreton, L. Marquer, N. Nebout-Combourieu, M. Stančikaitė, I. Tanţău, S. Tonkov, L. Shumilovskikh; LandClimII data contributors, European pollen-based REVEALS land-cover reconstructions for the Holocene: Methodology, mapping and potentials. *Earth Syst. Sci. Data* **14**, 1581–1619 (2022).

8. M. Horsák, N. Limondin-Lozouet, L. Juřičková, S. Granai, J. Horáčková, C. Legentil, V. Ložek, Holocene succession patterns of land snails across temperate Europe: East to west variation related to glacial refugia, climate and human impact. *Palaeogeogr. Palaeoclimatol. Palaeoecol.* **524**, 13–24 (2019).

9. C. J. Sandom, R. Ejrnæs, M. D. D. Hansen, J. C. Svenning, High herbivore density associated with vegetation diversity in interglacial ecosystems. *Proc. Natl. Acad. Sci. U.S.A.* **111**, 4162–4167 (2014).

10. H. M. Pereira, L. M. Navarro, *Rewilding European Landscapes* (Springer, 2015).

11. E. C. Ellis, N. Gauthier, K. K. Goldewijk, R. B. Bird, N. Boivin, S. Díaz, D. Q. Fuller, J. L. Gill, J. O. Kaplan, N. Kingston, H. Locke, C. N. H. McMichael, D. Ranco, T. C. Rick, M. R. Shaw, L. Stephens, J.-C. Svenning, J. E. M. Watson, People have shaped most of terrestrial nature for at least 12,000 years. *Proc. Natl. Acad. Sci. U.S.A.* **118**, e2023483118 (2021).

12. A. Nikulina, K. MacDonald, F. Scherjon, E. A. Pearce, M. Davoli, J. Svenning, E. Vella, M. Gaillard, A. Zapolska, F. Arthur, A. Martinez, K. Hatlestad, F. Mazier, M. A. Serge, K. Lindholm, R. Fyfe, H. Renssen, D. M. Roche, S. Kluiving, W. Roebroeks, Tracking hunter-gatherer impact on vegetation in last interglacial and Holocene Europe: Proxies and challenges. *J. Archaeol. Method Theory* **29**, 989–1033 (2022).

13. E. Dietze, M. Theuerkauf, K. Bloom, A. Brauer, W. Dörfler, I. Feeser, A. Feurdean, L. Gedminienė, T. Giesecke, S. Jahns, M. Karpińska-Kołaczek, P. Kołaczek, M. Lamentowicz, M. Latałowa, K. Marcisz, M. Obremska, A. Pędziszewska, A. Poska, K. Rehfeld, M. Stančikaitė, N. Stivrins, J. Święta-Musznicka, M. Szal, J. Vassiljev, S. Veski, A. Wacnik, D. Weisbrodt, J. Wiethold, B. Vannière, M. Słowiński, Holocene fire activity during low-natural flammability periods reveals scale-dependent cultural human-fire relationships in Europe. *Quat. Sci. Rev.* **201**, 44–56 (2018).

14. C. Sandom, S. Faurby, B. Sandel, J.-C. Svenning, Global late Quaternary megafauna extinctions linked to humans, not climate change. *Proc. R. Soc. B* **281**, 20133254 (2014).

15. E. S. Bakker, J. L. Gill, C. N. Johnson, F. W. Vera, C. J. Sandom, G. P. Asner, J.-C. Svenning, Combining paleo-data and modern exclosure experiments to assess the impact of megafauna extinctions on woody vegetation. *Proc. Natl. Acad. Sci. U.S.A.* **113**, 847–855 (2016).

16. F. A. Smith, R. E. E. Elliott Smith, S. K. Lyons, J. L. Payne, Body size downgrading of mammals over the late Quaternary. *Science* **360**, 310–313 (2018).

17. Y. Malhi, C. E. Doughty, M. Galetti, F. A. Smith, J. C. Svenning, J. W. Terborgh, Megafauna and ecosystem function from the Pleistocene to the Anthropocene. *Proc. Natl. Acad. Sci. U.S.A.* **113**, 838–846 (2016).

18. G. J. Kukla, M. L. Bender, J. de Beaulieu, G. Bond, W. S. Broecker, P. Cleveringa, J. E. Gavin, T. D. Herbert, J. Imbrie, J. Jouzel, L. D. Keigwin, K. Knudsen, J. F. McManus, J. Merkt, D. R. Muhs, H. Müller, R. Z. Poore, S. C. Porter, G. Seret, N. J. Shackleton, C. Turner, P. C. Tzedakis, I. J. Winograd, Last interglacial climates. *Quat. Res.* **58**, 2–13 (2002).

19. R. Dennell, Palaeoanthropology: Homo sapiens in China 80,000 years ago. *Nature* **526**, 647–648 (2015).

20. E. Pop, C. Bakels, Semi-open environmental conditions during phases of hominin occupation at the Eemian Interglacial basin site Neumark-Nord 2 and its wider environment. *Quat. Sci. Rev.* **117**, 72–81 (2015).

21. B. M. Benito, J.-C. Svenning, T. Kellberg-Nielsen, F. Riede, G. Gil-Romera, T. Mailund, P. C. Kjaergaard, B. S. Sandel, The ecological niche and distribution of Neanderthals during the Last Interglacial. *J. Biogeogr.* **44**, 51–61 (2017).

22. B. L. Otto-Bliesner, N. Rosenbloom, E. J. Stone, N. P. McKay, D. J. Lunt, E. C. Brady, J. T. Overpeck, How warm was the last interglacial? New model–data comparisons. *Philos. Trans. A Math. Phys. Eng. Sci.* **371**, 20130097 (2013).

23. K. J. Edwards, R. M. Fyfe, S. T. Jackson, The first 100 years of pollen analysis. *Nat. Plants.* **3**, 17001 (2017).

24. G. Lang, *Quartäre Vegetationsgeschichte Europas: Methoden und Ergebnisse* (G. Fischer Verlag Jena, 1994).

25. W. H. Zagwin, Vegetation, climate and radiocarbon datings in the Late Pleistocene of the Netherlands. Part 1: Eemian and Early Weichselian. *Med. Geol.* **14**, 15–58 (1961).

26. H. H. Birks, H. J. B. Birks, The rise and fall of forests. *Science* **305**, 484–485 (2004).

27. S. Sugita, Theory of quantitative reconstruction of vegetation I: Pollen from large sites REVEALS regional vegetation composition. *Holocene* **17**, 229–241 (2007).

28. A. Markova, A. Puzachenko, Preliminary analysis of European small mammal faunas of the Eemian interglacial: Species composition and species diversity at a regional scale. *Quaternary* **1**, 9 (2018).

29. I. C. Prentice, Pollen representation, source area, and basin size: Toward a unified theory of pollen analysis. *Quat. Res.* **23**, 76–86 (1985).

30. S. E. V. Hellman, M. Gaillard, A. Broström, S. Sugita, Effects of the sampling design and selection of parameter values on pollen-based quantitative reconstructions of regional vegetation: A case study in southern Sweden using the REVEALS model. *Veget. Hist. Archaeobot.* **17**, 445–459 (2008).

31. L. Marquer, F. Mazier, S. Sugita, D. Galop, T. Houet, E. Faure, M. Gaillard, S. Haunold, N. de Munnik, A. Simonneau, F. De Vleeschouwer, G. Le Roux, Pollen-based reconstruction of Holocene land-cover in mountain regions: Evaluation of the Landscape Reconstruction Algorithm in the Vicdessos valley, northern Pyrenees, France. *Quat. Sci. Rev.* **228**, 106049 (2020).

32. A.-K. Trondman, M. Gaillard, S. Sugita, L. Björkman, A. Greisman, T. Hultberg, P. Lagerås, M. Lindbladh, F. Mazier, Are pollen records from small sites appropriate for REVEALS model-based quantitative reconstructions of past regional vegetation? An empirical test in southern Sweden. *Veget. Hist. Archaeobot.* **25**, 131–151 (2016).

33. P. Kuneš, H. Svobodová-Svitavská, J. Kolář, M. Hajnalová, V. Abraham, M. Macek, P. Tkáč, P. Szabó, The origin of grasslands in the temperate forest zone of east-central Europe: Long-term legacy of climate and human impact. *Quat. Sci. Rev.* **116**, 15–27 (2015).

34. K. Stefaniak, R. Stachowicz-Rybka, R. K. Borówka, A. Hrynowiecka, A. Sobczyk, M. Moskal-del Hoyo, A. Kotowski, D. Nowakowski, M. T. Krajcarz, E. M. E. Billia, D. Persico, E. M. Burkanova, S. V. Leshchinskiy, E. van Asperen, U. Ratajczak, A. V. Shpansky, M. Lempart, B. Wach, M. Niska, J. van der Made, K. Stachowicz, J. Lenarczyk, J. Piątek, O. Kovalchuk, Browsers, grazers or mix-feeders? Study of the diet of extinct Pleistocene Eurasian forest rhinoceros Stephanorhinus kirchbergensis (Jäger,

1839) and woolly rhinoceros Coelodonta antiquitatis (Blumenbach, 1799). *Quat. Int.* **605–606**, 192–212 (2021).

35. T. van Kolfschoten, The Eemian mammal fauna of central Europe. *Neth. J. Geosci.* **79**, 269–281 (2000).

36. A. Bobiec, A. Reif, K. Öllerer, Seeing the oakscape beyond the forest: A landscape approach to the oak regeneration in Europe. *Landsc. Ecol.* **33**, 513–528 (2018).

37. A. M. Coppins, B. J. Coppins, Atlantic Hazelwoods – A neglected habitat? *Bot. J. Scotl.* **55**, 149–160 (2003).

38. C. Turner, Formal status and vegetational development of the Eemian interglacial in Northwestern and Southern Europe. *Quat. Res.* **58**, 41–44 (2002).

39. B. von Lüpke, Silvicultural methods of oak regeneration with special respect to shade tolerant mixed species. *Forest Ecol. Manag.* **106**, 19–26 (1998).

40. P. Kuneš, B. V. Odgaard, M.-J. Gaillard, Soil phosphorus as a control of productivity and openness in temperate interglacial forest ecosystems. *J. Biogeogr.* **38**, 2150–2164 (2011).

41. B. Woronko, Z. Zagórski, M. Cyglicki, Soil-development differentiation across a glacial–interglacial cycle, Saalian upland, E Poland. *CATENA* **211**, 105968 (2022).

42. K. Chytrý, W. Willner, M. Chytrý, J. Divíšek, S. Dullinger, Central European forest–steppe: An ecosystem shaped by climate, topography and disturbances. *J. Biogeogr.* **49**, 1006–1020 (2022).

43. E. Valdés-Correcher, E. Rodriguez, Y. J. M. Kemp, M. J. Wassen, J. P. G. M. Cromsigt, Comparing the impact of a grazing regime with European bison versus one with free-ranging cattle on coastal dune vegetation in the Netherlands. *Mamm. Res.* **63**, 455–466 (2018).

44. Ch. Leuschner, Resource availability at three presumed stages of a heathland succession on the Lüneburger Heide, Germany. *J. Veg. Sci.* **4**, 255–262 (1993).

45. M. Dvorský, O. Mudrák, J. Doležal, M. Jirků, Reintroduction of large herbivores restored plant species richness in abandoned dry temperate grassland. *Plant Ecol.* **223**, 525–535 (2022).

46. P. Cornelissen, J. Bokdam, K. Sykora, F. Berendse, Effects of large herbivores on wood pasture dynamics in a European wetland system. *Basic Appl. Ecol.* **15**, 396–406 (2014).

47. R. N. Owen-Smith, *Megaherbivores: The Influence of Very Large Body Size on Ecology* (Cambridge University Press, 2011).

48. C. Gao, D. H. Keen, S. Boreham, G Russell Coope, M. E. Pettit, A. J. Stuart, P. L. Gibbard, Last interglacial and Devensian deposits of the River Great Ouse at Woolpack Farm, Fenstanton, Cambridgeshire, UK. *Quat. Sci. Rev.* **19**, (787–810) (2000).

49. E. Berti, J.-C. Svenning, Megafauna extinctions have reduced biotic connectivity worldwide. *Glob. Ecol. Biogeogr.* **29**, 2131–2142 (2020).

50. M. Churski, T. Charles-Dominique, J. W. Bubnicki, B. Jędrzejewska, D. P. J. Kuijper, J. P. G. M. Cromsigt, Herbivore-induced branching increases sapling survival in temperate forest canopy gaps. *J. Ecol.* **110**, 1390–1402 (2022).

51. W. J. Bond, F. I. Woodward, G. F. Midgley, The global distribution of ecosystems in a world without fire. *New Phytol.* **165**, 525–538 (2005).

52. A. Feurdean, S. Tonkov, M. Pfeiffer, A. Panait, D. Warren, B. Vannière, E. Marinova, Fire frequency and intensity associated with functional traits of dominant forest type in the Balkans during the Holocene. *Eur. J. For. Res.* **138**, 1049–1066 (2019).

53. A. T. Karp, J. T. Faith, J. R. Marlon, A. C. Staver, Global response of fire activity to late Quaternary grazer extinctions. *Science* **374**, 1145–1148 (2021).

54. C. Bonavent, K. Olsen, R. Ejrnæs, C. Fløjgaard, M. D. D. Hansen, S. Normand, J.-C. Svenning, H. H. Bruun, Grazing by semi-feral cattle and horses supports plant species richness and uniqueness in grasslands. *Appl. Veg. Sci.* **26**, e12718 (2023).

55. M. Köhler, A. Schmidt, N. Hölzel, A. Baasch, S. Tischew, Positive long-term effects of year-round horse grazing in orchid-rich dry calcareous grasslands–Results of a 12-year study. *Front. Ecol. Evol.* **11**, 10.3389/fevo.2023.1107987 (2023).

56. M. S. Warren, D. Maes, C. A. M. van Swaay, P. Goffart, H. Van Dyck, N. A. D. Bourn, I. Wynhoff, D. Hoare, S. Ellis, The decline of butterflies in Europe: Problems, significance, and possible solutions. *Proc. Natl. Acad. Sci. U.S.A.* **118**, e2002551117 (2021).

57. A. Perino, H. M. Pereira, L. M. Navarro, N. Fernández, J. M. Bullock, S. Ceaușu, A. Cortés-Avizanda, R. van Klink, T. Kuemmerle, A. Lomba, G. Pe'er, T. Plieninger, J. M. Rey Benayas, C. J. Sandom, J. C. Svenning, H. C. Wheeler, Rewilding complex ecosystems. *Science* **364**, eaav5570 (2019).

58. B. Siegmar-Walter, *Walter's Vegetation of the Earth* (Springer, ed. 4, 2002).

59. S. Hellman, M.-J. Gaillard, A. Broström, S. Sugita, The REVEALS model, a new tool to estimate past regional plant abundance from pollen data in large lakes: Validation in southern Sweden. *J. Quat. Sci.* **23**, 21–42 (2008).

60. W. Soepboer, S. Sugita, A. F. Lotter, Regional vegetation-cover changes on the Swiss Plateau during the past two millennia: A pollen-based reconstruction using the REVEALS model. *Quat. Sci. Rev.* **29**, 472–483 (2010).

61. S. Sugita, T. Parshall, R. Calcote, K. Walker, Testing the Landscape Reconstruction Algorithm for spatially explicit reconstruction of vegetation in northern Michigan and Wisconsin. *Quat. Res.* **74**, 289–300 (2010).

62. M. A. Serge, F. Mazier, R. Fyfe, M.-J. Gaillard, T. Klein, A. Lagnoux, D. Galop, E. Githumbi, M. Mindrescu, A. B. Nielsen, A.-K. Trondman, A. Poska, S. Sugita, J. Woodbridge, D. Abel-Schaad, C. Åkesson, T. Alenius, B. Ammann, S. T. Andersen, R. S. Anderson, M. Andrič, L. Balakauskas, L. Barnekow, V. Batalova, J. Bergman, H. J. B. Birks, L. Björkman, A. E. Bjune, O. Borisova, N. Broothaerts, J. Carrion, C. Caseldine, J. Christiansen, Q. Cui, A. Currás, S. Czerwiński, R. David, A. L. Davies, R. De Jong, F. Di Rita, B. Dietre, W. Dörfler, E. Doyen, K. J. Edwards, A. Ejarque, E. Endtmann, D. Etienne, E. Faure, I. Feeser, A. Feurdean, E. Fischer, W. Fletcher, F. Franco-Múgica, E.

D. Fredh, C. Froyd, S. Garcés-Pastor, I. García-Moreiras, E. Gauthier, G. Gil-Romera, P. González-Sampériz, M. J. Grant, R. Grindean, J. N. Haas, G. Hannon, A.-J. Heather, M. Heikkilä, K. Hjelle, S. Jahns, N. Jasiunas, G. Jiménez-Moreno, I. Jouffroy-Bapicot, M. Kabailienė, I. M. Kamerling, M. Kangur, M. Karpińska-Kołaczek, A. Kasianova, P. Kołaczek, P. Lagerås, M. Latalowa, J. Lechterbeck, C. Leroyer, M. Leydet, M. Lindbladh, O. Lisitsyna, J.-A. López-Sáez, J. Lowe, R. Luelmo-Lautenschlaeger, E. Lukanina, L. Macijauskaitė, D. Magri, D. Marguerie, L. Marquer, A. Martinez-Cortizas, I. Mehl, J. M. Mesa-Fernández, T. Mighall, A. Miola, Y. Miras, C. Morales-Molino, A. Mrotzek, C. M. Sobrino, B. Odgaard, I. Ozola, S. Pérez-Díaz, R. P. Pérez-Obiol, C. Poggi, P. R. Rego, M. J. Ramos-Román, P. Rasmussen, M. Reille, M. Rösch, P. Ruffaldi, M. S. Goni, N. Savukynienė, T. Schröder, M. Schult, U. Segerström, H. Seppä, G. S. Vives, L. Shumilovskikh, H. W. Smettan, M. Stancikaite, A. C. Stevenson, N. Stivrins, I. Tantau, M. Theuerkauf, S. Tonkov, W. O. van der Knaap, J. F. N. van Leeuwen, E. Vecmane, G. Verstraeten, S. Veski, R. Voigt, H. Von Stedingk, M. P. Waller, J. Wiethold, K. J. Willis, S. Wolters, V. P. Zernitskaya, Testing the effect of relative pollen productivity on the REVEALS model: A validated reconstruction of Europe-Wide Holocene vegetation. *Land* **12**, 986 (2023).

63. F. Mazier, M.-J. Gaillard, P. Kuneš, S. Sugita, A.-K. Trondman, A. Broström, Testing the effect of site selection and parameter setting on REVEALS-model estimates of plant abundance using the Czech Quaternary Palynological Database. *Rev. Palaeobot. Palynol.* **187**, 38–49 (2012).

64. R. Hoevers, N. Broothaerts, G. Verstraeten, The potential of REVEALS-based vegetation reconstructions using pollen records from alluvial floodplains. *Veget Hist. Archaeobot.* **31**, 525–540 (2022).

65. J.-L. D. Beaulieu, M. Reille, A long Upper Pleistocene pollen record from les Echets, near Lyon, France. *Boreas* **13**, 111–132 (1984).

66. V. Abraham, V. Oušková, P. Kuneš, Present-day vegetation helps quantifying past land cover in selected regions of the Czech Republic. *PLOS ONE* **9**, e100117 (2014).

67. A. Stuart, J. K. Ord, *Kendall's Advanced Theory of Statistics, Distribution Theory* (John Wiley & Sons, 2010), vol. 1.

68. P. Scussolini, P. Bakker, C. Guo, C. Stepanek, Q. Zhang, P. Braconnot, J. Cao, M. V. Guarino, D. Coumou, M. Prange, P. J. Ward, H. Renssen, M. Kageyama, B. Otto-Bliesner, J. C. J. H. Aerts, Agreement between reconstructed and modeled boreal precipitation of the Last Interglacial. *Sci. Adv.* **5**, eaax7047 (2019).

69. X. Shi, H. Yang, C. Danek, G. Lohmann, *AWI AWI-ESM1.1LR Model Output Prepared for CMIP6 PMIP* (*Earth System Grid Federation*, 2020) (1 March 2022).

70. A. Voldoire, D. Saint-Martin, S. Sénési, B. Decharme, A. Alias, M. Chevallier, J. Colin, J.-F. Guérémy, M. Michou, M.-P. Moine, P. Nabat, R. Roehrig, D. Salas y Mélia, R. Séférian, S. Valcke, I. Beau, S. Belamari, S. Berthet, C. Cassou, J. Cattiaux, J. Deshayes, H. Douville, C. Ethé, L. Franchistéguy, O. Geoffroy, C. Lévy, G. Madec, Y. Meurdesoif, R. Msadek, A. Ribes, E. Sanchez-Gomez, L. Terray, R. Waldman, Evaluation of CMIP6 DECK experiments with CNRM-CM6-1. *J. Adv. Model. Earth Syst.* **11**, 2177–2213 (2019).

71. M. Kelley, G. A. Schmidt, L. S. Nazarenko, S. E. Bauer, R. Ruedy, G. L. Russell, A. S. Ackerman, I. Aleinov, M. Bauer, R. Bleck, V. Canuto, G. Cesana, Y. Cheng, T. L. Clune, B. I. Cook, C. A. Cruz, A. D. Del Genio, G. S. Elsaesser, G. Faluvegi, N. Y. Kiang, D. Kim, A. A. Lacis, A. Leboissetier, A. N. LeGrande, K. K. Lo, J. Marshall, E. E. Matthews, S. McDermid, K. Mezuman, R. L. Miller, L. T. Murray, V. Oinas, C. Orbe, C. P. García-Pando, J. P. Perlwitz, M. J. Puma, D. Rind, A. Romanou, D. T. Shindell, S. Sun, N. Tausnev, K. Tsigaridis, G. Tselioudis, E. Weng, J. Wu, M. S. Yao, Giss-E2.1: Configurations and climatology. *J. Adv. Model. Earth Syst.* **12**, e2019MS002025 (2020).

72. E. Volodin, E. Mortikov, A. Gritsun, V. Lykossov, V. Galin, N. Diansky, A. Gusev, S. Kostrykin, N. Iakovlev, A. Shestakova, S. Emelina, *INM INM-CM4–8 Model Output Prepared for CMIP6 CMIP piControl (Earth System Grid Federation*, 2019) (1 March 2022).

73. O. Boucher, J. Servonnat, A. L. Albright, O. Aumont, Y. Balkanski, V. Bastrikov, S. Bekki, R. Bonnet, S. Bony, L. Bopp, P. Braconnot, P. Brockmann, P. Cadule, A. Caubel, F. Cheruy, F. Codron, A. Cozic, D. Cugnet, F. D'Andrea, P. Davini, C. de Lavergne, S. Denvil, J. Deshayes, M. Devilliers, A. Ducharne, J. Dufresne, E. Dupont, C. Éthé, L. Fairhead, L. Falletti, S. Flavoni, M. Foujols, S. Gardoll, G. Gastineau, J. Ghattas, J. Grandpeix, B. Guenet, L. E. Guez, E. Guilyardi, M. Guimberteau, D. Hauglustaine, F. Hourdin, A. Idelkadi, S. Joussaume, M. Kageyama, M. Khodri, G. Krinner, N. Lebas,

G. Levavasseur, C. Lévy, L. Li, F. Lott, T. Lurton, S. Luyssaert, G. Madec, J. Madeleine, F. Maignan, M. Marchand, O. Marti, L. Mellul, Y. Meurdesoif, J. Mignot, I. Musat, C. Ottlé, P. Peylin, Y. Planton, J. Polcher, C. Rio, N. Rochetin, C. Rousset, P. Sepulchre, A. Sima, D. Swingedouw, R. Thiéblemont, A. K. Traore, M. Vancoppenolle, J. Vial, J. Vialard, N. Viovy, N. Vuichard, Presentation and evaluation of the IPSL-CM6A-LR climate model. *J. Adv. Model. Earth Syst.* **12**, e2019MS002010 (2020).

74. T. Hajima, M. Watanabe, A. Yamamoto, H. Tatebe, M. A. Noguchi, M. Abe, R. Ohgaito, A. Ito, D. Yamazaki, H. Okajima, A. Ito, K. Takata, K. Ogochi, S. Watanabe, M. Kawamiya, Development of the Miroc-ES2L Earth system model and the evaluation of biogeochemical processes and feedbacks. *Geosci. Model Dev.* **13**, 2197–2244 (2020).

75. D. N. Karger, O. Conrad, J. Böhner, T. Kawohl, H. Kreft, R. W. Soria-Auza, N. E. Zimmermann, H. P. Linder, M. Kessler, Climatologies at high resolution for the earth's land surface areas. *Sci. Data* **4**, 170122 (2017).

76. S. E. Fick, R. J. Hijmans, WorldClim 2: New 1-km spatial resolution climate surfaces for global land areas. *Int. J. Climatol.* **37**, 4302–4315 (2017).

77. H. E. Beck, N. E. Zimmermann, T. R. McVicar, N. Vergopolan, A. Berg, E. F. Wood, Present and future Köppen-Geiger climate classification maps at 1-km resolution. *Sci. Data* **5**, 180214 (2018).

78. P. C. Tzedakis, R. N. Drysdale, V. Margari, L. C. Skinner, L. Menviel, R. H. Rhodes, A. S. Taschetto, D. A. Hodell, S. J. Crowhurst, J. C. Hellstrom, A. E. Fallick, J. O. Grimalt, J. F. McManus, B. Martrat, Z. Mokeddem, F. Parrenin, E. Regattieri, K. Roe, G. Zanchetta, Enhanced climate instability in the North Atlantic and southern Europe during the Last Interglacial. *Nat. Commun.* **9**, 4235 (2018).

79. S. Normand, R. E. Ricklefs, F. Skov, J. Bladt, O. Tackenberg, J.-C. Svenning, Postglacial migration supplements climate in determining plant species ranges in Europe. *Proc. R. Soc. B Biol. Sci.* **278**, 3644–3653 (2011).

80. S. Muff, E. B. Nilsen, R. B. O'Hara, C. R. Nater, Rewriting results sections in the language of evidence. *Trends Ecol. Evol.* **37,** 203–210 (2022).

81. A.-K. Trondman, M.-J. Gaillard, F. Mazier, S. Sugita, R. Fyfe, A. B. Nielsen, C. Twiddle, P. Barratt, H. J. B. Birks, A E Bjune, L. Björkman, A. Broström, C. Caseldine, R. David, J. Dodson, W. Dörfler, E. Fischer, B van Geel, T. Giesecke, T. Hultberg, L. Kalnina, M. Kangur, P. van der Knaap, T. Koff, P. Kuneš, P. Lagerås, M. Latałowa, J. Lechterbeck, C. Leroyer, M. Leydet, M. Lindbladh, L. Marquer, F. J. G. Mitchell, B. V. Odgaard, S. M. Peglar, T. Persson, A. Poska, M. Rösch, H. Seppä, S. Veski, L. Wick, Pollen-based quantitative reconstructions of Holocene regional vegetation cover (plant-functional types and land-cover types) in Europe suitable for climate modelling. *Glob. Chang. Biol.* **21**, 676–697 (2015).

82. C. Prentice, "Records of vegetation in time and space: The principles of pollen analysis" in *Vegetation History, Handbook of Vegetation Science*. B. Huntley, T. Webb, Eds. (Springer Netherlands, 1988), pp. 17–42.

83. P. Bakker, H. Renssen, Last Interglacial model–data mismatch of thermal maximum temperatures partially explained. *Clim. Past* **10**, 1633–1644 (2014).

84. L. Marks, M. Makos, M. Szymanek, B. Woronko, J. Dzierżek, A. Majecka, Late Pleistocene climate of Poland in the mid-European context. *Quat. Int.* **504**, 24–39 (2019).

85. C. M. Nicholson, Eemian paleoclimate zones and Neanderthal landscape-use: A GIS model of settlement patterning during the last interglacial. *Quat. Int.* **438**, 144–157 (2017).

86. P. González-Sampériz, G. Gil-Romera, E. García-Prieto, J. Aranbarri, A. Moreno, M. Morellón, M. Sevilla-Callejo, M. Leunda, L. Santos, F. Franco-Múgica, A. Andrade, J. S. Carrión, B. L. Valero-Garcés, Strong continentality and effective moisture drove unforeseen vegetation dynamics since the last interglacial at inland Mediterranean areas: The Villarquemado sequence in NE Iberia. *Quat. Sci. Rev.* **242**, 106425 (2020).

87. C. Gao, S. Boreham, Ipswichian (Eemian) floodplain deposits and terrace stratigraphy in the lower Great Ouse and Cam valleys, southern England, UK. *Boreas* **40**, 303–319 (2011).

88. A. Schnitzler, Towards a new European wilderness: Embracing unmanaged forest growth and the decolonisation of nature. *Landsc. Urban Plan.* **126**, 74–80 (2014).

89. F. Cribari-Neto, A. Zeileis, Beta Regression in R. *J. Stat. Softw.* **34**, 1–24 (2010).

90. J. C. Douma, J. T. Weedon, Analysing continuous proportions in ecology and evolution: A practical introduction to beta and Dirichlet regression. *Methods Ecol. Evol.* **10**, 1412–1430 (2019).

91. S. Boreham, K. Leszczynska, The Geology of the Middle Cam Valley, Cambridgeshire UK. *Quaternary* **2**, 24 (2019).

92. W. H. Zagwijn, Vegetation, climate and radiocarbon datings in the Late Pleistocene of the Netherlands. *Meded. Geol. Sticht.* **14**, 15–45 (1961).

93. P. Cleveringa, T. Meijer, R. J. W. van Leeuwen, H. de Wolf, R. Pouwer, T. Lissenberg, A. W. Burger, The Eemian stratotype locality at Amersfoort in the central Netherlands: A re-evaluation of old and new data. *Geol. Mijnb./Neth.* **79**, 197–216(2000).

94. W. Ricken, E. Grüger, Vegetationsentwicklung, Paläoböden, Seespiegelschwankungen: Untersuchungen an eem- und weichselzeitlichen Sedimenten vom Südrand des Harzes. *EGQSJ.* **38**, 37–51 (1988).

95. R. J. W. van Leeuwen, D. J. Beets, J. H. A. Bosch, A. W. Burger, P. Cleveringa, D. van Harten, G. F. W. Herngreen, R. W. Kruk, C. G. Langereis, T. Meijer, R. Pouwer, H. de Wolf, Stratigraphy and integrated facies analysis of the Saalian and Eemian sediments in the Amsterdam-Terminal borehole, the Netherlands. *Geol. Mijnb./Neth. J.* **79**, 161–196 (2000).

96. C. Kasse, J. D. van der Woude, H. A. G. Woolderink, J. Schokker, Eemian to Early Weichselian regional and local vegetation development and sedimentary and geomorphological controls, Amersfoort Basin, The Netherlands. *Geol. Mijnb./Neth. J.* **101**, e7 (2022).

97. A. Börner, A. Hrynowiecka, V. Kuznetsov, R. Stachowicz-Rybka, F. Maksimov, V. Grigoriev, M. Niska, M. Moskal-del Hoyo, Palaeoecological investigations and 230Th/U dating of Eemian interglacial peat sequence of Banzin (Mecklenburg-Western Pomerania, NE-Germany). *Quat. Int.* **386**, 122–136 (2015).

98. A. Hrynowiecka, R. Stachowicz-Rybka, M. Niska, M. Moskal-del Hoyo, A. Börner, H. Rother, Eemian (MIS 5e) climate oscillations based on palaeobotanical analysis from the Beckentin profile (NE Germany). *Quat. Int.* **605–606**, 38–54 (2021).

99. M. Malkiewicz, Palynology of biogenic sediments of the Eemian Interglacial at Bieganin near Kalis Central Poland. *Geol. Q.* **47**, 367–372 (2003).

100. H. Müller, Pollenanalytische Untersuchungen und Jahresschichtenzälung an der eem-zeitlichen Kieselgur von Bispingen/Luhe. *Geol. Jahrb.* **21**, 149–169 (1974).

101. N. Hermsdorf, J. Strahl, Eemian deposits in the Brandenburg area Brandenburg. *Geowiss. Beitr.* **15**, 23–55 (2008).

102. J. Schokker, P. Cleveringa, A. S. Murray, Palaeoenvironmental reconstruction and OSL dating of terrestrial Eemian deposits in the southeastern Netherlands. *J. Quat. Sci.* **19**, 193–202 (2004).

103. M. Malkiewicz, Pollen-based vegetation and climate reconstruction of the Eemian sequence from Buntowo, N Poland. *Quat. Int.* **467**, 54–61 (2018).

104. O. K. Borisova, Vegetation and climate changes at the Eemian/Weichselian transition: New palynological data from Central Russian Plain. *Pol. Geol. Inst. Spec. Pap.* **16**, 9–17 (2005).

105. K. Bińka, J. Nitychoruk, Cyclicity in the Eemian climate? A case study of the Eemian site at Czaple, Eastern Poland. *Rev. Palaeobot. Palynol.* **164**, 39–44 (2011).

106. D. H. Keen, M. D. Bateman, G. R. Coope, M. H. Field, H. E. Langford, J. S. Merry, T. M. Mighall, Sedimentology, palaeoecology and geochronology of Last Interglacial deposits from Deeping St James, Lincolnshire. *J. Quat. Sci*. **14**, 411–436 (1999).

107. W. De Gans, The Drentsche Aa valley system (Vrije Universiteit Te Amsterdam, 1981).

108. K. Bińka, J. Nitychoruk, The Late Saalian, Eemian and Early Vistulian pollen sequence at Dziewule, eastern Poland. *Geol. Q.* **47**, 155–168 (2003).

109. H-J. Beug, Vegetationsgeschichtliche-pollenanalytische Untersuchungen am Riß/Würm-Interglazial von Eurach am Starnberger See/Obb. *Geolog. Bavarica* **80**, 91–106 (1979).

110. J. Mangerud, H.-P. Sejrup, E. Sønstegaard, S. Haldorsen, A continuous Eemian-Early Weichselian sequence containing pollen and marine fossils at Fjøsanger, western Norway. *Boreas* **10**, 137–208 (1981).

111. U. C. Müller, J. Pross, E. Bibus, Vegetation response to rapid climate change in Central Europe during the past 140,000 yr based on evidence from the Füramoos pollen record. *Quat. Res.* **59**, 235–245 (2003).

112. J. Niklewski, Interglacial eemski w Glowczynie kolo Wyszogrodu. *Monogr. Bot.* **27**, 125–191 (1968).

113. Z. Janczyk-Kopikowa, Interglacjal eemski w Golkowie kolo Warszawy. *Kwart. Geol.* **10**, 453–461 (1966).

114. S. Wegmüller, Recherches palynologiques sur les charbons feuilletés de la région de Gondiswil/Ufhusen (plateau suisse). *Quaternaire* **23**, 29–34 (1986).

115. K-E. Behre, Pollen- und diatomeenanalytische Untersuchungen an letztinterglazialen Kieselgurlagern der Lüneburger Heide: Schwindebeck und Grevenhof im oberen Luhetal. *Flora oder Allgemeine Botanische Zeitung* **152**, 325–370 (1962).

116. L. Eissmann, T. Litt, The Saalian sequence in the type region (Central Germany). *INQUA Subcommission on European Quarternary Strutigraphy*, **58**, Halle (1992).

117. M. Malkiewicz, The history of vegetation of the Eemian Interglacial in the Great Polish Lowland. *Acta Soc. Bot. Pol.* **71**, 311–321 (2002).

118. M. Malkiewicz, Early Vistulian vegetation history and climate change at Gutów (Wielkopolska Lowland) from pollen analysis. *Geol. Q.* **54**, 357–366 (2010).

119. J. Strahl, Detailergebnisse pollenanalytischer Untersuchungen an saalespätglazialen bis weichselfrühglazialen Sedimenten aus dem Kiestagebau Hinterste Mühle bei Neubrandenburg (Mecklenburg-Vorpommern). *Brandenb. Geowiss. Beitr* **7**, 2 (2000).

120. S. T. Andersen, Interglacial vegetational succession and lake development in Denmark. *Palaeobotanist* **15**, 117–127 (1966).

121. W. Granoszewski, Late Pleistocene vegetation history and climatic changes at Horoszki Duże, eastern Poland: A palaeobotanical study. *Acta Palaeobot.* **4**, 3–95 (2003).

122. K. Mamakowa, Late Middle Polish Glaciation, Eemian and Early Vistulian vegetation at Imbramowice near Wrocław and the pollen stratigraphy of this part of the Pleistocene in Poland. *Acta Palaeobot.* **29**, 11–176 (1989).

123. P. C. Tzedakis, M. R. Frogley, T. H. E. Heaton, Last Interglacial conditions in southern Europe: Evidence from Ioannina, northwest Greece. *Glob. Planet. Change.* **36**, 157–170 (2003).

124. J. Rychel, M. T. Karasiewicz, I. Krześlak, L. Marks, B. Noryśkiewicz, B. Woronko, Paleogeography of the environment in north-eastern Poland recorded in an Eemian sedimentary basin, based on the example of the Jałówka site. *Quat. Int.* **328-329**, 60–73 (2014).

125. U. C. Müller, A Late-Pleistocene pollen sequence from the Jammertal, south-western Germany with particular reference to location and altitude as factors determining Eemian forest composition. *Veg. Hist. Archaebot.* **9**, 125–131 (2000).

126. A.-M. Robertsson, L. Rodhe, A Late Pleistocene sequence at Seitevare Swedish Lapland. *Boreas* **17**, 501–509 (1988).

127. K. Erd, Pollenanalytische Untersuchungen im Pleistozän der DDR. *Abh. Zentr. Geol. Inst.* **18**, 1–7 (1973).

128. M. Żarski, A new locality of Eemian Interglacial deposits near Dęblin. *Kwart. Geol.* **33**, 269–274 (1989).

129. B. Frenzel, “Über einen frühen letzteiszeitlichen Vorstoß des Rheingletschers in das deutsche Alpenvorland” in *Klimageschichtliche Probleme der letzten 130,000 Jahre Paläoklimaforschung*, B. Frenzel, Ed. (Gustav Fischer Verlag Stuttgart, 1991), pp. 377–400.

130. B. Noryśkiewicz, Analiza palinologiczna osadów organicznych ze stanowiska Kwiatków Las. Szczegółowa Mapa Geologiczna Polski, 1: 50 000, arkusz Skalmierzyce. Mat. Arch. Zak. Geomorf. Univ. Łódz. (1995).

131. G. J. Kukla, J.-L. de Beaulieu, H. Svobodova, V. Andrieu-Ponel, N. Thouveny, H. Stockhausen, Tentative correlation of pollen records of the last interglacial at Grande Pile and Ribains with marine isotope stages. *Quatern. Res.* **58**, 32–35 (2002).

132. M. Reille, V. Andrieu, J. De beaulieu, P. Guenet, C. Goeury, A long pollen record from Lac du Bouchet, Massif Central, France: For the period Ca. 325 to 100 ka bp (OIS 9c to OIS 5e). *Quat. Sci. Rev.* **17**, 1107–1123 (1998).

133. N. Pickarski, O. Kwiecien, M. Djamali, T. Litt, Vegetation and environmental changes during the last interglacial in eastern Anatolia (Turkey): A new high-resolution pollen record from Lake Van. *Palaeogeogr. Palaeoclimatol. Palaeoecol.* **435**, 145–158 (2015).

134. Z. Balwierz, M. Roman, A new Eemian interglacial to Early Vistulian site at Łanięta, central Poland. *Geol. Q.* **46**, 207–217 (2002).

135. J. Lundqvist, The interglacial deposit at the Leveäniemi mine, Svappavaara, Swedish Lapland (Sveriges reproduktions AB (distr.), 1971).

136. K. M. Krupinski, Pollen profile LOM2-78, Lomzyca Poland. *European Pollen Database* 10.1594/PANGAEA.711922 (2009).

137. V. Šeirienė, N. Kühl, D. Kisielienė, Quantitative reconstruction of climate variability during the Eemian (Merkinė) and Weichselian (Nemunas) in Lithuania. *Quatern. Res.* **82**, 229–235 (2014).

138. W. Stankowski, M. Nita, Stratigraphy of Late Quaternary deposits and their neotectonic record in the Konin area Central Poland. *Geol. Q.* **48,** 23–24 (2004)

139. J. R. M. Allen, U. Brandt, A. Brauer, H.-W. Hubberten, B. Huntley, J. Keller, M. Kraml, A. Mackensen, J. Mingram, J. F. W. Negendank, N. R. Nowaczyk, H. Oberhänsli, W. A. Watts, S. Wulf, B. Zolitschka, Rapid environmental changes in southern Europe during the last glacial period. *Nature* **400,** 740–743 (1999).

140. B. Noryśkiewicz, Interglaciał eemski w Nakle nad Notecią. *Acta Palaeobot.* **19**, 67–112 (1978)

141. C. Bakels, Non-pollen palynomorphs from the Eemian pool Neumark-Nord 2: Determining water quality and the source of high pollen-percentages of herbaceous taxa. *Rev. Palaeobot. Palynol.* **186,** 58–61 (2012).

142. K. Bińka, J. Nitychoruk, J. Dzierżek, Climate stability during the Eemian – New pollen evidence from the Nidzica site, northern Poland. *Boreas.* **40,** 342–350 (2011).

143. G. Lemdahl, A. Broström, L. Hedenäs, K. Arvidsson, S. Holmgren, M.-J. Gaillard, P. Möller, Eemian and Early Weichselian environments in southern Sweden: A multi-proxy study of till-covered organic deposits from the Småland peneplain. *J. Quat. Sci.* **28**, 705–719 (2013).

144. K.-E. Behre, J. van der Plicht, Towards an absolute chronology for the last glacial period in Europe: Radiocarbon dates from Oerel, northern Germany. *Veg. Hist. Archaeobot.* **1**, 111–117 (1992).

145. K-E. Behre, K. Göttlich, J. Werner, *Die Vegetation im Spätpleistozän von Osterwanna, Niedersachsen* (Bundesanstalt für Bodenforschung, 1974).

146. H. Klatkowa, H. Winter, The Eemian interglacial in Ostrow near Grabica. *Acta Geogr. Lodz.* **61,** 59–68 (1990).

147. O. K. Borisova, E. Y. Novenko, A. A. Velichko, K. V. Kremenetski, F. W. Junge, T. Boettger, Vegetation and climate changes during the Eemian and Early Weichselian in the Upper Volga region (Russia). *Quat. Sci. Rev.* **26**, 2574–2585 (2007).

148. K. Urbański, H. Winter, Stanowisko interglacjału eemskiego w Radówku (Pojezierze Łagowskie, zachodnia Polska) i jego implikacje dla litostratygrafii glin zwałowych. *Prz. Geol.* **53**, 418–424 (2005).

149. R. Kühner, J. Strahl, Eemian deposits at the maximum glacial extent of the Warthian stage in the open cast lignite mine Welzow-Sud, Niederlausitz. *Z. Dt. Ges. Geowiss.* **159**, 191–204 (2008).

150. J.-L. de Beaulieu, M. Reille, Long Pleistocene pollen sequences from the Velay Plateau (Massif Central, France). *Veg. Hist. Archaeobot.* **1**, 233–242 (1992).

151. H. Winter, E. Dobracka, D. Ciszek, Multidyscyplinarne badania osadów eemskich i wczesnovistuliańskich z profilu Rzecino (Wysoczyzna Łobeska, Pojezierze Zachodniopomorskie). *Biul. Państw. Inst. Geol.* **428**, 93–109 (2008).

152. E. Grüger, Spätriß, Riß/Würm und Frühwürm am Samerberg in Oberbayern – Ein vegetationsgeschichtlicher Beitrag zur Gliederung des Jungpleistozäns. *Geol. Bavarica*, **80**, 5–64 (1979).

153. K. Erd, Vegetationsentwicklung und Pollenanalysen im Eem-Interglazial und Weichsel-Frühglazial von Schönfeld, Kreis Calau. *Natur und Landschaft in der Niederlausitz*, **1,** 71–81 (1991).

154. B. Urban, H. Elsner, A. Hölzer, D. Mania, B. Albrecht, Eine eem- und frühweichselzeitliche Abfolge im Tagebau Schöningen, Landkreis Helmstedt. *EGQSJ.* **41**, 85–99 (1991).

155. K-E. Behre, Pollen- und diatomeenanalytische Untersuchungen an letztinterglazialen Kieselgurlagern der Lüneburger Heide. *Flora order Allgemeine Botanische Zeitung* **152**, 325–370.e3 (1962).

156. C. Verbruggen, Quaternary palaeobotanical evolution of Northern Belgium. *Geol. Belg.* **2**, 99–110 (1999).

157. W. Stankowski, A. Bluszcz, M. Nita, "Stanowiska osadów górnoczwartorzędowych Mikorzyn i Sławoszewek w świetle badań geologicznych, datowania radiowęglowego i luminescencyjnego oraz analiz palinologicznych" in *Geochronologia górnego czwartorzędu Polski w świetle datownia radiowęglowego i luminescencyjnego*, A. Pazur, W. Bluszcz, L. Stankowski, L. Starkel, Eds. (Wydawnictwo Instytutu Fizyki Politechniki Śląskiej, Gliwice), pp. 87–111.

158. J. S. Salonen, K. F. Helmens, J. Brendryen, N. Kuosmanen, M. Väliranta, S. Goring, M. Korpela, M. Kylander, A. Philip, A. Plikk, H. Renssen, M. Luoto, Abrupt high-latitude climate events and decoupled seasonal trends during the Eemian. *Nat. Commun.* **9**, 2851 (2018).

159. P. Schläfli, E. Gobet, J. F. N. van Leeuwen, E. Vescovi, M. A. Schwenk, D. Bandou, G. A. Douillet, F. Schlunegger, W. Tinner, Palynological investigations reveal Eemian interglacial vegetation dynamics at Spiezberg, Bernese Alps, Switzerland. *Quat. Sci. Rev.* **263**, 106975 (2021).

160. C. Martin, G. Ménot, N. Thouveny, N. Davtian, V. Andrieu-Ponel, M. Reille, E. Bard, Impact of human activities and vegetation changes on the tetraether sources in Lake St Front (Massif Central, France). *Org. Geochem.* **135**, 38–52 (2019).

161. M. Malkiewicz, A Late Saalian Glaciation, Eemian Interglacial and Early Weichselian pollen sequence at Szklarka, SW Poland – Reconstruction of vegetation and climate. *Quat. Int.* **467**, 43–53 (2018).

162. A. M. Milner, K. H. Roucoux, R. E. L. Collier, U. C. Müller, J. Pross, P. C. Tzedakis, Vegetation responses to abrupt climatic changes during the Last Interglacial Complex (Marine Isotope Stage 5) at Tenaghi Philippon, NE Greece. *Quat. Sci. Rev.* **154**, 169–181 (2016).

163. F. Sirocko, K. Seelos, K. Schaber, B. Rein, F. Dreher, M. Diehl, R. Lehne, K. Jäger, M. Krbetschek, D. Degering, A late Eemian aridity pulse in central Europe during the last glacial inception. *Nature* **436**, 833–836 (2005).

164. P. Kołaczek, M. Karpińska-Kołaczek, J. Petera-Zganiacz, Vegetation patterns under climate changes in the Eemian and Early Weichselian in Central Europe inferred from a palynological sequence from Ustków (central Poland). *Quat. Int.* **268**, 9–20 (2012).

165. M. Follieri, D. Magri, L. Sadori, Pollen stratigraphical synthesis from Valle di Castiglione (Roma). *Quat. Int.* **3-4**, 81–84 (1989).

166. C. Lüthgens, M. Böse, T. Lauer, M. Krbetschek, J. Strahl, D. Wenske, Timing of the last interglacial in Northern Europe derived from Optically Stimulated Luminescence (OSL) dating of a terrestrial Saalian–Eemian–Weichselian sedimentary sequence in NE-Germany. *Quat. Int.* **241**, 79–96 (2011).

167. K. M. Krupinski, W. Morawsk, Geological Position and Pollen Analysis of Eemian Interglacial Sediments of Warsaw - Wawrzyszew. *Acta Palaeobot.* **33**, 309–346 (1993).

168. A. Hall, Some new palaeobotanical records for the British Ipswichian Interglacial. *New Phytol.* **81**, 805–812 (1978).

169. I. A. Pidek, S. Terpiłowski, Osady organogeniczne eemskie i wczesnovistuliańskie w Wiśniewie koło Siedlec. *Ann. UMCS B.* **48**, 229–238 (1993).

170. E. Grüger, A. Schreiner, Riß/Würm- und würmzeitliche Ablagerungen im Wurzacher Becken (Rheingletschergebiet). *Neues Jahrb. für Geol. Paläontol.* **189**, 81–117 (1993).
